# Two decades of malaria control in Malawi: Geostatistical Analysis of the changing malaria prevalence from 2000-2022

**DOI:** 10.12688/wellcomeopenres.19390.1

**Published:** 2023-06-21

**Authors:** Donnie Mategula, Judy Gichuki, Michael Give Chipeta, James Chirombo, Patrick Ken Kalonde, Austin Gumbo, Michael Kayange, Vincent Samuel, Colins Kwizombe, Gracious Hamuza, Alinafe Kalanga, Dina Kamowa, Colins Mitambo, Jacob Kawonga, Benard Banda, Jacob Kafulafula, Akuzike Banda, Halima Twabi, Esloyn Musa, Maclear Masambuka, Tapiwa Ntwere, Chimwemwe Ligomba, Lumbani Munthali, Melody Sakala, Abdoulaye Bangoura, Atupele Kapito-Tembo, Nyanyiwe Masingi-Mbeye, Don P. Mathanga, Dianne J Terlouw

**Affiliations:** 1 School of Global and Public Health, Kamuzu University of Health Sciences, Blantyre, Southern, Malawi; 2 Malawi-Liverpool Wellcome Programme, Blantyre, Malawi; 3 Liverpool School of Tropical Medicine, Liverpool, L35QA, UK; 4 Strathmore University, Institute of Healthcare Management, Nairobi, Kenya; 5 African Institute for Development Policy (AFIDEP), Lilongwe, Malawi; 6 National Malaria Control Programme, Ministry of Health, Lilongwe, Malawi; 7 U.S. President's Malaria Initiative, United States Agency for International Development (USAID), Lilongwe, Malawi; 8 Mulanje District Council, Mulanje, Malawi; 9 Research Unit, Ministry of Health, Lilongwe, Malawi; 10 Country Health Information Systems and Data Use (CHISU) Program, Lilongwe, Malawi; 11 Nkhotakota District Council, Nkhotakota, Malawi; 12 Department of Mathematical Sciences, School of Natural and Applied Sciences, University of Malawi, Zomba, Malawi; 13 Kasungu District Council, Kasungu, Malawi; 14 PMI VectorLink Project, Abt Associates,, Lilongwe, Malawi

**Keywords:** Model-based geostatistics, malaria, Malawi, Plasmodium falciparum

## Abstract

*Background*

Malaria remains a public health problem in Malawi and has a serious socio-economic impact on the population. In the past two decades, available malaria control measures have been substantially scaled up, such as insecticide-treated bed nets, artemisinin-based combination therapies, and, more recently, the introduction of the malaria vaccine, the RTS,S/AS01. In this paper, we describe the epidemiology of malaria for the last two decades to understand the past transmission and set the scene for the elimination agenda.

*Methods*

A collation of parasite prevalence surveys conducted between the years 2000 and 2022 was done. A spatio-temporal geostatistical model was fitted to predict the yearly malaria risk for children aged 2–10 years (PfPR 2–10) at 1×1 km spatial resolutions. Parameter estimation was done using the Monte Carlo maximum likelihood methods. District level prevalence estimates adjusted for population are calculated for the years 2000 to 2022.

*Results*

A total of 2,595 sampled unique locations from 2000 to 2022 were identified through the data collation exercise. This represents 70,565 individuals that were sampled in the period. In general, the PfPR2_10 declined over the 22 years. The mean modeled national PfPR2_10 in 2000 was 43.93 % (95% CI:17.9 to 73.8%) and declined to 19.2% (95%CI 7.49 to 37.0%) in 2022. The smoothened estimates of PfPR2_10 indicate that malaria prevalence is very heterogeneous with hotspot areas concentrated on the southern shores of Lake Malawi and the country's central region.

*Conclusions*

The last two decades are associated with a decline in malaria prevalence, highly likely associated with the scale up of control interventions. The country should move towards targeted malaria control approaches informed by surveillance data.

## Introduction

Malaria is a disease of public health importance affecting many communities to date ^1^. In 2019, it was estimated that there were 215 million malaria cases globally, 94% of which were in sub-Saharan Africa ^2^. Malaria infection is caused by protozoa of the genus Plasmodium that has five known species that are responsible for human infection ^3^: Plasmodium falciparum, P. vivax, P. ovale, and P. malariae, more recently, P. knowlesi,. The vector responsible for human transmission is the female anopheles mosquito ^4^. In Sub-Saharan Africa, malaria is mainly caused by Plasmodium falciparum and is one of the leading causes of morbidity and mortality especially in children under five years ^5^. Other high-risk groups include pregnant women ^6^ and immunologically naïve persons like travelers coming from non-endemic places ^7^.

In 2016, The World Health Organisation (WHO) released the global technical strategy (GTS) for malaria 2016–2030 to help countries accelerate progress toward malaria elimination. The strategy targets reducing global malaria incidence and mortality rates by at least 90% by 2030 and eliminating in at least 35 countries by 2030 ^8^. Malawi has aligned its malaria strategic goals to GTS, and the country reflected its commitment to the GTS in the 2016–2022 Malaria strategic plan ^9^ and will plan to continue implementing the strategy beyond 2022.

Since 2010, malaria control efforts in Malawi have scaled up substantially through multiple control measures that include bednets, Artemisinin Combination Therapies (ACTs) and malaria Rapid Diagnostic Tests (mRDTs). With this, malaria transmission has reduced by 44% ^10, 11^, and clinical case data has become much more accurate as over 95% of government-provided treatments are now based on mRDT results ^12^. As malaria transmission declines, its heterogeneity will increase, and transmission will be increasingly driven from 'hotspots.' At lower transmission levels, targeted control efforts becomes essential to maximize available resources' impact and further reduce the burden ^13^. In 2019, the Malawi National Malaria Control Programme (NMCP) was on track against its Malaria Strategic Plan (MSP) for 2017–2022 to reduce malaria incidence by 50% by 2022 from an initial baseline of 386 per 1,000 population in 2015 (2019 NMCP programme review). If progress continues, sub-district targeted control will become crucial for the next seven-years (MSP 2023–2030).

The Malawi NMCP priorities are in line with WHO guidance for countries to regularly analyse their key malaria indicators to predict, respond, and monitor the malaria situation in-country in terms of intervention delivery, coverage, and disease burden. This includes the ability to detect local malaria hotspots to guide control programmes with timely evidence-informed responses ^8^. Malaria risk mapping has a long legacy in Africa, including Malawi. Mosquito breeding site maps became available in the 1950s, soon after the discovery of mosquitos as the malaria vector by Sir Ronald Ross ^14^. Early European settlers did early risk maps for Malawi in their attempts to provide cartographic information on climate, agriculture, and mosquito breeding sites. These maps provided control agencies with a plan for larval control, environmental management, and mass drug administration targets ^15^.

Application of the more recent methods of model-based geostatistical (MBG) methods to map malaria risk in Malawi only started to be used in 2006. Kazembe and colleagues in their work utilised data on malaria prevalence from 73 sampled survey locations, where children aged 1–10 years had been sampled between 1970 and 2001 ^16^. Their work was directly used in the Malawi malaria programme review in 2010 and the national strategic plan 2011–2015 to highlight the hyper-endemic nature of malaria transmission in the country, with variations in higher altitude areas.

Efforts to model spatio-temporal heterogeneity in malaria have focused on parasite prevalence (infection) data from household surveys, because of concerns over the quality and completeness of routine clinical malaria case data from the district health information system (DHIS2). In 2019, Chipeta and colleagues used MBG methods to describe the changing malaria transmission in Malawi between the years 2010 and 2017 ^17^.

There have been several exciting developments in malaria control in the period between 2017 and 2021. In 2019, the country started piloting the RTS,S malaria vaccine in 11 districts. The RTS,S/AS01 is a leading malaria vaccine candidate developed to prevent diseases caused by Plasmodium falciparum. The results of a phase 3 trial of this vaccine confirmed moderate protection with overall efficacy estimates of 46% (95% CI 42,50) against clinical malaria and 38% (95% CI 18, 53) against severe malaria by 18 months after dose 3. A fourth dose, given 18 months after dose 3, increased efficacy against clinical malaria from 26% (95% CI 21, 31) to 39% (95% CI 34,43) and from -2 (95% CI - 31,20) to 31.5% (95% CI 9,48) against severe malaria. Vaccine efficacy was also confirmed against malaria hospitalization (37%, 95%CI 27-48.5), all-cause hospitalization (15%, 95%CI 6-25) and severe anaemia (62%, 95%CI 26.5-81) in children who received 4 doses of RTS, S/AS01 ^18, 19^. Following a successful pilot, the WHO has recommended the vaccine for wider use. Another success has been the mass net distribution campaigns. In 2018, the country introduced piperonyl butoxide (PBO) net in addition to the regular long-lasting insecticide treated bed nets ^20^. Additionally, Malawi also implemented annual indoor residual spraying (IRS) in some districts in 2018 and annually afterwards.

The same period has been faced with several challenges: The COVID-19 pandemic reduced the health care seeking for most febrile illnesses including malaria. There were also associated health system disturbances because of the pandemic. Other health system disruptions included tropical storms in 2019 and 2021, measles outbreak in 2017, polio outbreak in 2021 and cholera outbreak in 2020.

Carrying forward the successes in the last five years, the NMCP is poised to develop the revised malaria strategic plan (MSP) in 2023–2030. Informed by strengthened surveillance systems, the NMCP is transitioning from blanket interventions across the country to exploring options for targeted intervention strategies. This is already reflected in the five-year integrated vector control strategy (IVCS) plan for the period 2020–2024 that informs net distribution and targeted indoor residual spraying (IRS). Similarly, the NMCP is evaluating the expansion of community-level malaria case management in over five year olds for hard-to-reach and high burden areas. Targeted interventions will become a key part of the 2022–2027 MSP, which is scheduled to be developed in 2022/2023. It is for this reason why it is important to describe the malaria epidemiology in both space and time. The aim of this analysis is to describe the malaria epidemiology prevalence for the last two decades including description of the subnational disease risk to guide target interventions.

## Methods

### Study area

Malawi is a small country in sub-Saharan Africa. It neighbours Tanzania in the north, Mozambique in the south, southwest and east and Zambia to the west. The country has five major lakes (Lakes Malawi, Malombe, Chilwa, Chiuta, Kazuni and Kaulime) that contribute to 21% of the country's 118,484 km ^2^ territorial surface area. The country consists of four main geographical regions: the Great Rift Valley, the central plateaus, the highlands, and the isolated mountains with the rift valley forming the most striking topographic feature that runs for the entire length of the narrow country. It passes through Lake Malawi in the Northern and Central Regions and stretches to the Shire Valley in the south. This contributes to the variable temperature patterns in Malawi that average 144° to 32° Celsius based on altitude and proximity to the lake ^17, 21^. There are three weather seasons hot-wet, hot-dry and cool-dry. From May to August, the weather is cool and dry, becomes hot in September and October, and the rainy season begins in October or November, continuing until April.

**Figure 1.**
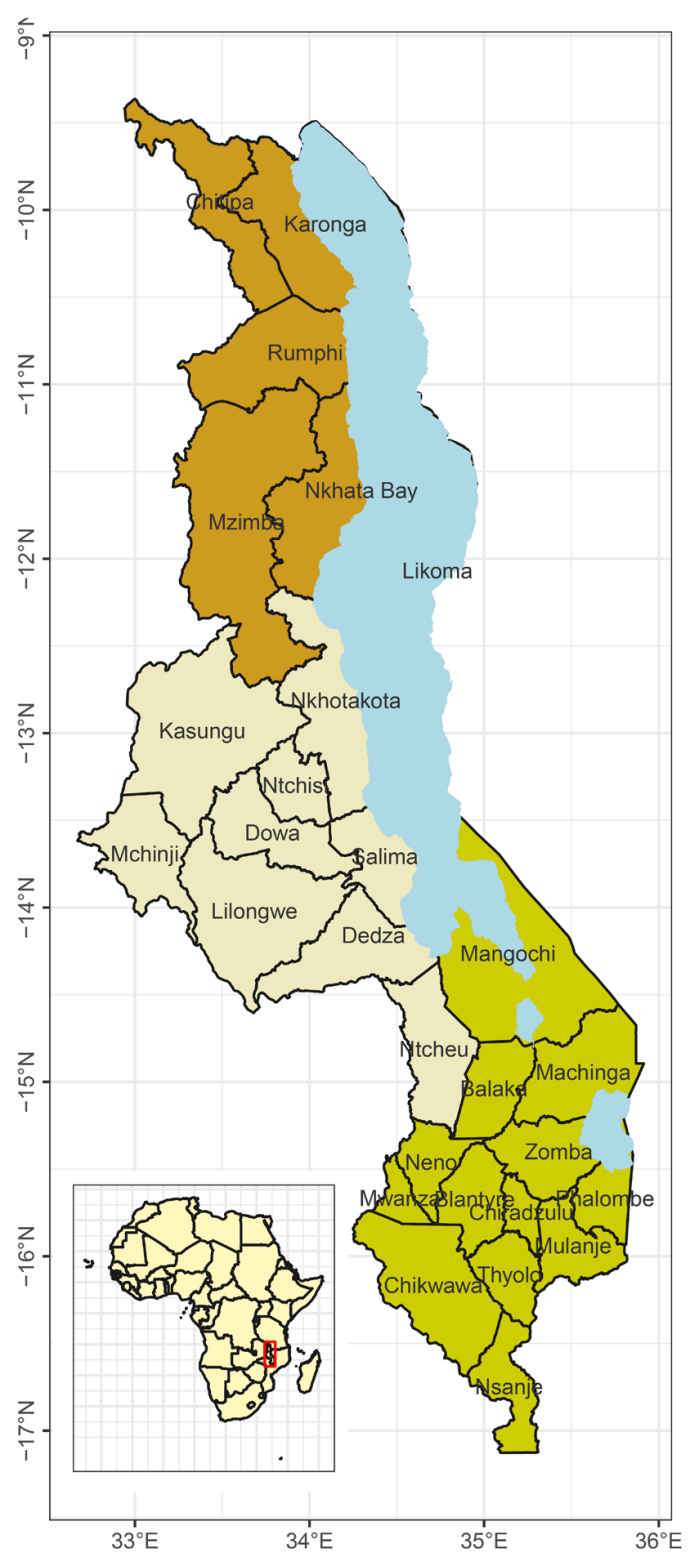
Map of Malawi showing the 28 districts of Malawi.

The country is divided into three regions namely Northern, Central, and Southern regions. There are 28 districts in the country: 6 districts in the Northern region, 9 in the Central region, and 13 in the Southern region ( Figure 1). The country has a population of 17.6 million ^18, 22^ and a GDP per capita of 320 USD.

### Data gathering

The collation of malaria survey data into a single geocoded repository followed a cascaded approach. The first step followed a more traditional peer-reviewed publication search in various data bases: PubMed, Google Scholar, the WHO Library Database and African Journals Online. The key words used for this search were "malaria" and "Malawi". The last electronic search was completed in June 2022. The next step was a data request from the Malawi NMCP for the national Malaria indicator survey data for the surveys done in 2010, 2012, 2015, 2017 and 2021. The last step was to reach out to the research community within Malawi for any unpublished survey data. This included data collected as part of the malaria vaccine pilot implementation programme.

From each of the identified survey reports the minimum required data fields for each record were: date and location, age range information about blood examination (number of individuals tested and number positive for Plasmodium infections by species), the methods used to detect and, the lowest and highest age in the surveyed population (decimal years).

### Model description

The spatio-temporal variation in PfPR2-10 was modeled using geostatistical methods to borrow strength of information across time and space.

Let *Y _i_* denote the number of individuals that test positive for plasmodium falciparum at survey cluster location *x _i_* and time *t _i_*

And that the survey team went to the sampled clusters given by *x _i_* and sampled *m _i_*: *i* = 1…. *n* at time *t* individuals at risk in the cluster and recorded the outcome of every person that tests positive and negative for plasmodium falciparum malaria.

Then standard geostatistical spatial-temporal model, then assumes that:


$$Y_{i}\sim Binomial\;(m_{i},P(x_{i},\;t_{i})$$

*Y _i_* is a Binomial distribution with *m _i_* trials and probability of a positive test *P*( *x _i_*, *t _i_*) specified in the binomial geostatistical model below:


$$log\left\{\frac{PfPR(x,t)}{1-PfPR(x,t)}\right\}=\alpha +\beta *mA+\gamma *MA+TSI(x,\;t)+S(x,\;t)+Z(x,\;t)\text{,}$$

where *mA* and *MA* are the min and max age among the sampled individuals at location *x*. TSI represents temperature suitability index at location x and time t. S(x,t) to denote the variation in malaria risk between communities (e.g. variation due to different other environmental conditions) and Z(x,t) the variation within communities (i.e. genetic and behavioural traits). In statistical jargon, S(x,t) and Z(x,t) are so-called random effects that are used in a model in order to capture the effects of unmeasured malaria risk factors. A stationary and isotropic Gaussian process for the spatio-temporal random effects is assumed S(x, t), with an exponential correlation function given as


cor{S(x,t),S(x^',t^')}=e^{-‖u‖/φ}e^{|-v|/Ψ}

where φ and ψ are scale parameters which regulate the rate of decay of the spatial and temporal correlation for the increasing distance and time separation, respectively; u = ||x − x^'|| is the distance in space between the location of any two communities, one at x and the other at x^'; ν = |t − t^'| is the time separation in years between any two surveys.

The model parameters were estimated via maximum likelihood in the R software environment (version 3.4.1) using logit-transformed prevalence. The targets for the predictions were PfPR2-10 over the 1 x 1 km regular grid surface covering the whole of Malawi. Maps of malaria risk were generated for the years 2000–2022 in QGIS Version 3.2

Uncertainty of the prevalence estimates was addressed using the traditional approach of confidence intervals generated from standard errors of the estimates. Additionally, a more novel approach of *Exceedance Probabilities* was explored. It has been argued that *Exceedance Probabilities*, could be an approach that is more relevant to policy makers, than the traditional approach of using confidence intervals. *Exceedance Probabilities (E.P.)* method sets policy relevant thresholds, that sets the probability that the prevalence is exceeds a policy relevant threshold. The *E.P.* can be formally expressed as:


$$EP=Probability\{,P(x_{i})>t|\text{data}\}$$

where *t* is the prevalence threshold. This approach in this work was only exploratory and do not present it in this paper

### Model validation

To test whether there was any evidence against spatial correlation in the data, empirical variogram methods was used. A simulation of 1000 empirical variograms around the fitted model were ran and these were used to compute 95% confidence intervals at any given spatial distance of the variogram. A conclusion was reached that there is spatial correlation in the data if the empirical variogram obtained from the data fell outside the 95% tolerance bandwidth.

### Ethical considerations

This is a secondary data analysis, and therefore exempt from ethical approval. The original study participants from which the data was obtained consented to participate in the surveys. The data used in the analysis was collated such that identity or exact location of the human subjects could not be ascertained directly or through identifiers linked to the subjects. Permission to use the dataset was obtained from the either the investigators, or from the National Malaria Control Programme, through the set data request procedures for the institutions.

## Results

**Figure 2.**
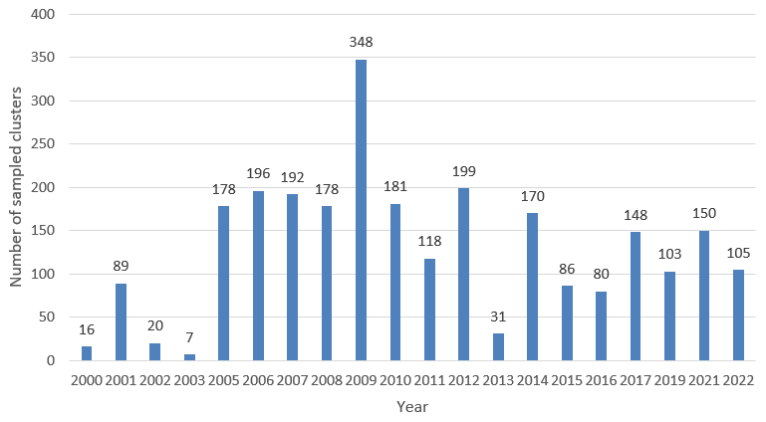
Distribution of samples sites/clusters by year.

A total of 2,595 sampled unique locations from 2000 to 2022 were identified through the data collation exercise. This represents 70,565 individuals that were sampled in the period. The distribution of the sampled locations across the years is shown in Figure 2 below.

**Figure 3.**
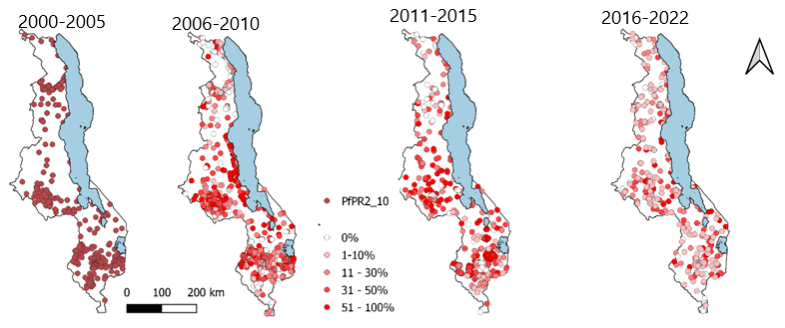
Spatial temporal distribution of the sampled locations 2000–2022.

The sampled locations were distributed across the entire surface of Malawi as shown in Figure 3 below.

There was a decline in the prevalence of malaria ages 2-10(PfPR2_10) in the sampled locations over the period 2000 to 2022. The period 2000 to 2005 was associated with sampled locations having higher prevalence which was followed by points having a reducing PfPR2_10. The year 2021 has the greatest number of locations with the least PfPR2_10.

**Figure 4.**
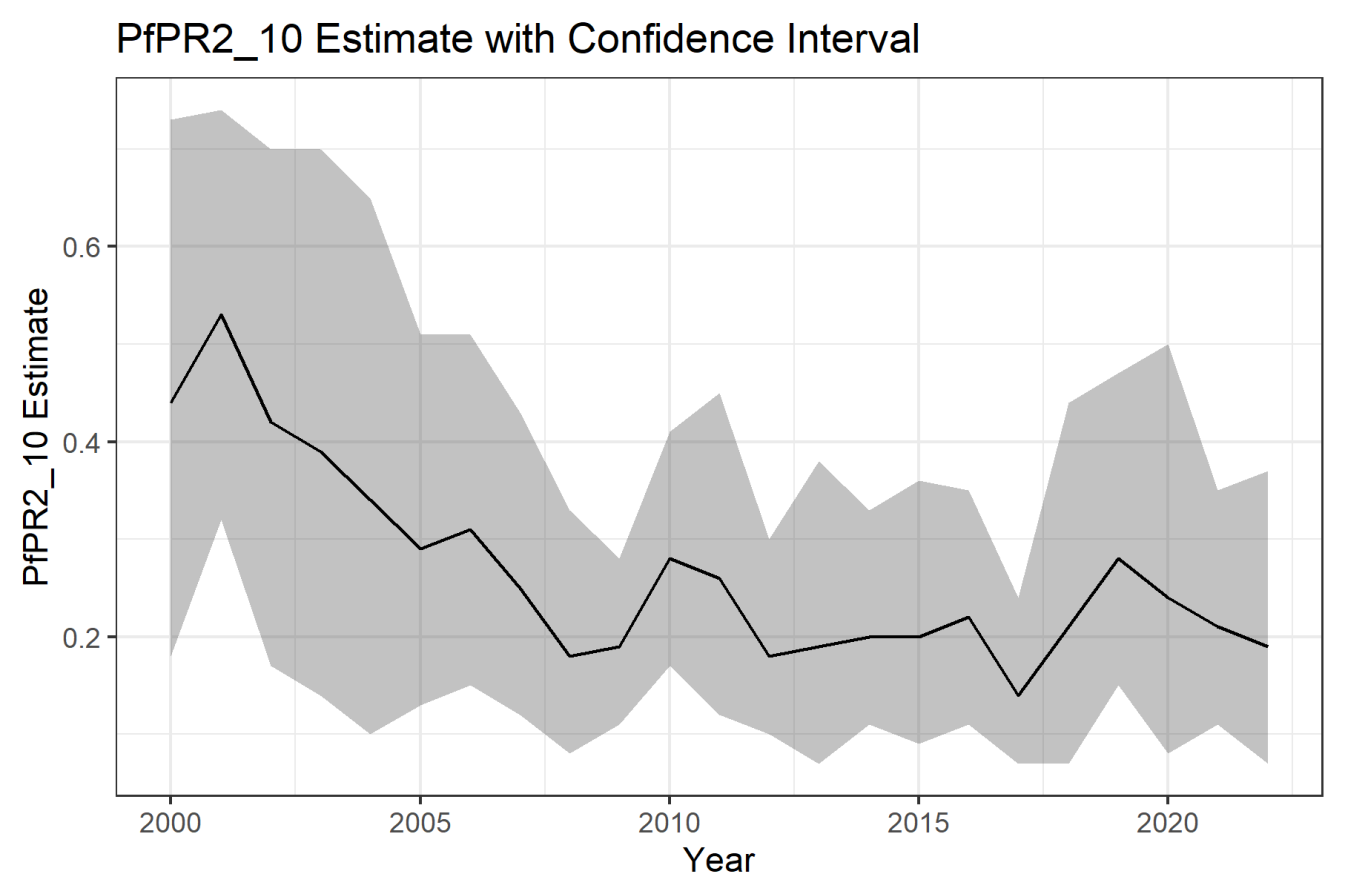
National PfPR 2-10 2000–2022.

In general, the PfPR2_10 was declining over the 22 years. The mean modelled national PfPR2_10 in 2000 was 43.93% (95%CI: 17.92%-72.84) and declined in the subsequent years. Table 1 below shows the national PfPR2_10 estimates with their associated confidence intervals (CI). Figure 4 below shows the national PFPR2_10 estimates and their associated 95% confidence intervals for the years 2000 to 2022. The same estimates are presented in Table 1 below.

**Table 1.** Modelled National PfPR2_10 for the years 2000–2022.

	Lower CI	PfPR2_10 Estimate	Upper CI
2000	17.92%	43.93%	72.84%
2001	31.77%	53.35%	74.26%
2002	16.61%	42.05%	70.31%
2003	13.72%	39.37%	70.21%
2004	10.44%	33.87%	64.96%
2005	12.92%	29.30%	51.26%
2006	15.36%	30.88%	50.74%
2007	12.15%	24.93%	43.23%
2008	8.35%	18.45%	33.26%
2009	11.32%	18.54%	28.38%
2010	16.88%	27.56%	40.97%
2011	11.98%	26.10%	45.31%
2012	9.71%	18.20%	29.96%
2013	6.96%	19.17%	37.72%
2014	10.88%	20.28%	33.40%
2015	8.56%	19.55%	35.93%
2016	11.47%	21.65%	35.40%
2017	6.93%	13.81%	23.92%
2018	6.56%	20.84%	43.52%
2019	14.54%	28.39%	47.26%
2020	7.64%	24.40%	49.94%
2021	10.58%	20.68%	34.53%
2022	7.49%	19.23%	37.05%

**Figure 5.**
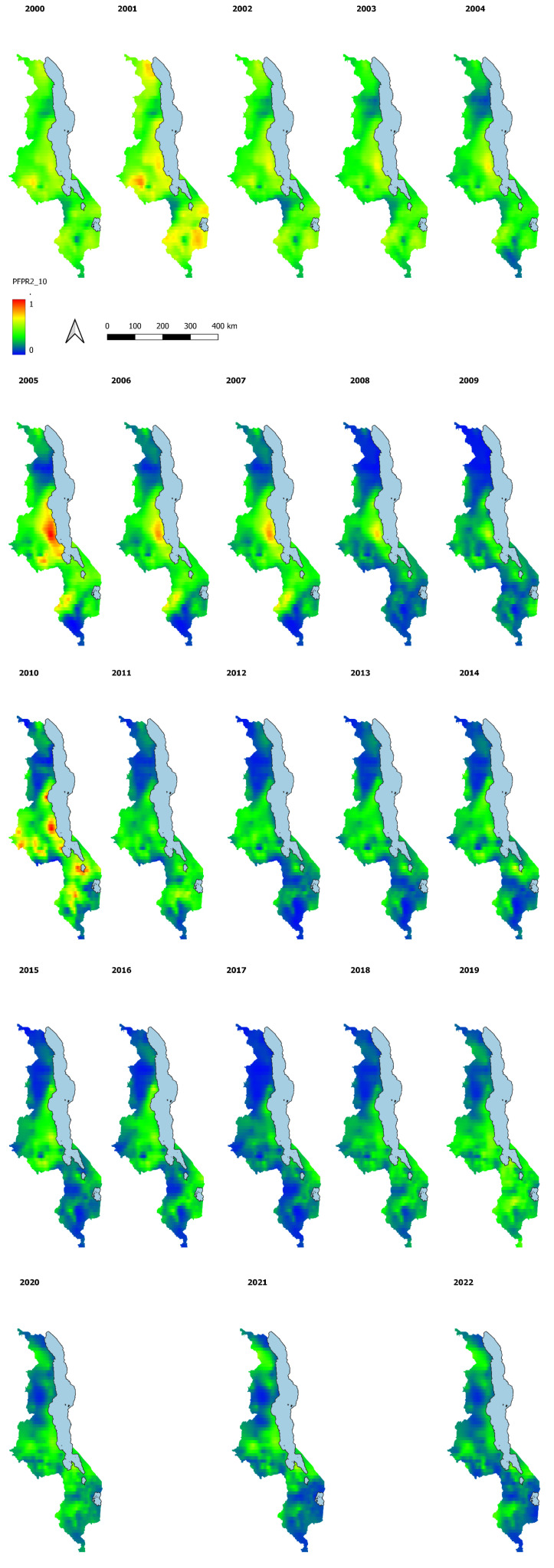
PfPR 2_10 at 1x1Km resolution years 2000 to 2022.

While we show the national estimates above, the main objective of this analysis is to map the malaria prevalence estimates and their associated uncertainty for the whole surface of Malawi at higher resolution (1x1 Km grids and district level) for the period of interest as these estimates are not available with the traditional MIS. The maps in Figure 5 below indicate the modelled PfPR2_10 at a spatial resolution of 1km by 1 km grid. The predictions have been made for the years 2000 to 2022.

The model outputs confirm the heterogeneous nature of malaria transmission in Malawi, with central and southern lake shore areas having higher PfPR2_10 than other parts of the country. Comparison of the high-resolution maps shows the spatial-temporal decline in malaria prevalence especially in Northern parts of Malawi across the years.

**Figure 6.**
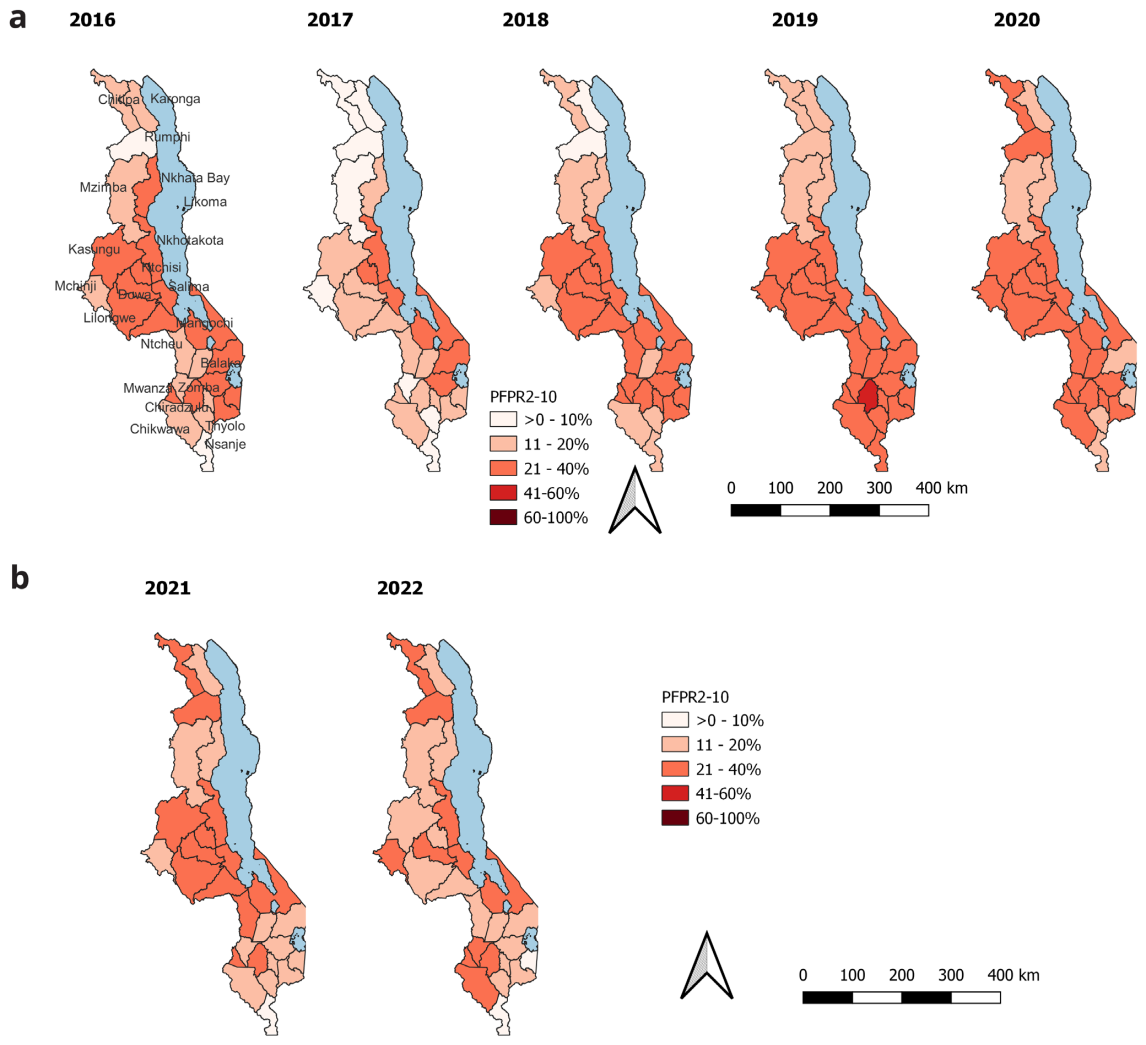
District PfPR2_10 from 2016–2022.

Malawi has a decentralized health system, where decision making is at district level. For that reason, we present district level prevalence estimates for the period 2016 to 2022, that coincides with the most recent strategic plan. These estimates are shown in the map in Figure 6 bellow. There is remarkable decline in PfPR2_10 in 2017 as compared to the previous years. Higher malaria transmission is in the districts on the central southern tip of Lake Malawi over the years, though the prevalence has been decreasing. In 2021, there was an increase in PfPR2_10 in some districts. This include one district (Chitipa) in the north and seven districts in the central region, with prevelence estimates in the 11–20% category. District level PfPR estimates for the period 2000 to 2022 are shown in Table 2.

**Table 2.** District level PfPR2-10 for the years 2000 to 2022.

	District	year	estimate	lowerCI	upperCI
1	Balaka	2000	0.46708	0.46708	0.46708
2	Balaka	2001	0.577297	0.577297	0.577297
3	Balaka	2002	0.421669	0.421669	0.421669
4	Balaka	2003	0.424598	0.424598	0.424598
5	Balaka	2004	0.381258	0.381258	0.381258
6	Balaka	2005	0.346026	0.346026	0.346026
7	Balaka	2006	0.406241	0.406241	0.406241
8	Balaka	2007	0.218141	0.218141	0.218141
9	Balaka	2008	0.183419	0.183419	0.183419
10	Balaka	2009	0.165954	0.165954	0.165954
11	Balaka	2010	0.37501	0.37501	0.37501
12	Balaka	2011	0.255785	0.255785	0.255785
13	Balaka	2012	0.123459	0.123459	0.123459
14	Balaka	2013	0.145745	0.145745	0.145745
15	Balaka	2014	0.213248	0.213248	0.213248
16	Balaka	2015	0.144601	0.144601	0.144601
17	Balaka	2016	0.14117	0.14117	0.14117
18	Balaka	2017	0.104721	0.104721	0.104721
19	Balaka	2018	0.189773	0.189773	0.189773
20	Balaka	2019	0.292796	0.292796	0.292796
21	Balaka	2020	0.201223	0.201223	0.201223
22	Balaka	2021	0.12319	0.12319	0.12319
23	Balaka	2022	0.127611	0.127611	0.127611
24	Blantyre	2000	0.371898	0.371898	0.371898
25	Blantyre	2001	0.530028	0.530028	0.530028
26	Blantyre	2002	0.389533	0.389533	0.389533
27	Blantyre	2003	0.406776	0.406776	0.406776
28	Blantyre	2004	0.338562	0.338562	0.338562
29	Blantyre	2005	0.266709	0.266709	0.266709
30	Blantyre	2006	0.266432	0.266432	0.266432
31	Blantyre	2007	0.15504	0.15504	0.15504
32	Blantyre	2008	0.132585	0.132585	0.132585
33	Blantyre	2009	0.153468	0.153468	0.153468
34	Blantyre	2010	0.420646	0.420646	0.420646
35	Blantyre	2011	0.411004	0.411004	0.411004
36	Blantyre	2012	0.168474	0.168474	0.168474
37	Blantyre	2013	0.167375	0.167375	0.167375
38	Blantyre	2014	0.168022	0.168022	0.168022
39	Blantyre	2015	0.164971	0.164971	0.164971
40	Blantyre	2016	0.234759	0.234759	0.234759
41	Blantyre	2017	0.129548	0.129548	0.129548
42	Blantyre	2018	0.258528	0.258528	0.258528
43	Blantyre	2019	0.425417	0.425417	0.425417
44	Blantyre	2020	0.312122	0.312122	0.312122
45	Blantyre	2021	0.209605	0.209605	0.209605
46	Blantyre	2022	0.278373	0.278373	0.278373
47	Chikwawa	2000	0.471119	0.471119	0.471119
48	Chikwawa	2001	0.565085	0.565085	0.565085
49	Chikwawa	2002	0.439608	0.439608	0.439608
50	Chikwawa	2003	0.399554	0.399554	0.399554
51	Chikwawa	2004	0.262118	0.262118	0.262118
52	Chikwawa	2005	0.164383	0.164383	0.164383
53	Chikwawa	2006	0.18315	0.18315	0.18315
54	Chikwawa	2007	0.148868	0.148868	0.148868
55	Chikwawa	2008	0.127093	0.127093	0.127093
56	Chikwawa	2009	0.186133	0.186133	0.186133
57	Chikwawa	2010	0.25585	0.25585	0.25585
58	Chikwawa	2011	0.226446	0.226446	0.226446
59	Chikwawa	2012	0.1256	0.1256	0.1256
60	Chikwawa	2013	0.192291	0.192291	0.192291
61	Chikwawa	2014	0.232918	0.232918	0.232918
62	Chikwawa	2015	0.156084	0.156084	0.156084
63	Chikwawa	2016	0.189644	0.189644	0.189644
64	Chikwawa	2017	0.118396	0.118396	0.118396
65	Chikwawa	2018	0.199495	0.199495	0.199495
66	Chikwawa	2019	0.287659	0.287659	0.287659
67	Chikwawa	2020	0.227101	0.227101	0.227101
68	Chikwawa	2021	0.159569	0.159569	0.159569
69	Chikwawa	2022	0.210868	0.210868	0.210868
70	Chiradzulu	2000	0.37823	0.37823	0.37823
71	Chiradzulu	2001	0.57739	0.57739	0.57739
72	Chiradzulu	2002	0.411002	0.411002	0.411002
73	Chiradzulu	2003	0.39966	0.39966	0.39966
74	Chiradzulu	2004	0.303495	0.303495	0.303495
75	Chiradzulu	2005	0.188744	0.188744	0.188744
76	Chiradzulu	2006	0.186006	0.186006	0.186006
77	Chiradzulu	2007	0.086475	0.086475	0.086475
78	Chiradzulu	2008	0.100149	0.100149	0.100149
79	Chiradzulu	2009	0.124891	0.124891	0.124891
80	Chiradzulu	2010	0.307661	0.307661	0.307661
81	Chiradzulu	2011	0.386074	0.386074	0.386074
82	Chiradzulu	2012	0.108813	0.108813	0.108813
83	Chiradzulu	2013	0.095221	0.095221	0.095221
84	Chiradzulu	2014	0.077174	0.077174	0.077174
85	Chiradzulu	2015	0.107677	0.107677	0.107677
86	Chiradzulu	2016	0.194292	0.194292	0.194292
87	Chiradzulu	2017	0.103756	0.103756	0.103756
88	Chiradzulu	2018	0.202838	0.202838	0.202838
89	Chiradzulu	2019	0.325055	0.325055	0.325055
90	Chiradzulu	2020	0.248765	0.248765	0.248765
91	Chiradzulu	2021	0.173388	0.173388	0.173388
92	Chiradzulu	2022	0.190959	0.190959	0.190959
93	Chitipa	2000	0.459803	0.459803	0.459803
94	Chitipa	2001	0.550883	0.550883	0.550883
95	Chitipa	2002	0.432906	0.432906	0.432906
96	Chitipa	2003	0.336376	0.336376	0.336376
97	Chitipa	2004	0.266253	0.266253	0.266253
98	Chitipa	2005	0.199763	0.199763	0.199763
99	Chitipa	2006	0.180517	0.180517	0.180517
100	Chitipa	2007	0.166822	0.166822	0.166822
101	Chitipa	2008	0.072805	0.072805	0.072805
102	Chitipa	2009	0.036958	0.036958	0.036958
103	Chitipa	2010	0.148754	0.148754	0.148754
104	Chitipa	2011	0.11549	0.11549	0.11549
105	Chitipa	2012	0.081175	0.081175	0.081175
106	Chitipa	2013	0.099548	0.099548	0.099548
107	Chitipa	2014	0.101955	0.101955	0.101955
108	Chitipa	2015	0.07838	0.07838	0.07838
109	Chitipa	2016	0.111941	0.111941	0.111941
110	Chitipa	2017	0.065219	0.065219	0.065219
111	Chitipa	2018	0.11435	0.11435	0.11435
112	Chitipa	2019	0.167884	0.167884	0.167884
113	Chitipa	2020	0.223861	0.223861	0.223861
114	Chitipa	2021	0.284936	0.284936	0.284936
115	Chitipa	2022	0.261515	0.261515	0.261515
116	Dedza	2000	0.414943	0.414943	0.414943
117	Dedza	2001	0.447592	0.447592	0.447592
118	Dedza	2002	0.315216	0.315216	0.315216
119	Dedza	2003	0.339062	0.339062	0.339062
120	Dedza	2004	0.352674	0.352674	0.352674
121	Dedza	2005	0.375278	0.375278	0.375278
122	Dedza	2006	0.349513	0.349513	0.349513
123	Dedza	2007	0.203309	0.203309	0.203309
124	Dedza	2008	0.166374	0.166374	0.166374
125	Dedza	2009	0.19755	0.19755	0.19755
126	Dedza	2010	0.198574	0.198574	0.198574
127	Dedza	2011	0.186091	0.186091	0.186091
128	Dedza	2012	0.173095	0.173095	0.173095
129	Dedza	2013	0.205162	0.205162	0.205162
130	Dedza	2014	0.244009	0.244009	0.244009
131	Dedza	2015	0.28182	0.28182	0.28182
132	Dedza	2016	0.264483	0.264483	0.264483
133	Dedza	2017	0.154764	0.154764	0.154764
134	Dedza	2018	0.236234	0.236234	0.236234
135	Dedza	2019	0.305433	0.305433	0.305433
136	Dedza	2020	0.253332	0.253332	0.253332
137	Dedza	2021	0.200068	0.200068	0.200068
138	Dedza	2022	0.140879	0.140879	0.140879
139	Dowa	2000	0.481649	0.481649	0.481649
140	Dowa	2001	0.554933	0.554933	0.554933
141	Dowa	2002	0.43189	0.43189	0.43189
142	Dowa	2003	0.392477	0.392477	0.392477
143	Dowa	2004	0.363323	0.363323	0.363323
144	Dowa	2005	0.333291	0.333291	0.333291
145	Dowa	2006	0.325107	0.325107	0.325107
146	Dowa	2007	0.338098	0.338098	0.338098
147	Dowa	2008	0.218177	0.218177	0.218177
148	Dowa	2009	0.195398	0.195398	0.195398
149	Dowa	2010	0.283432	0.283432	0.283432
150	Dowa	2011	0.287564	0.287564	0.287564
151	Dowa	2012	0.330185	0.330185	0.330185
152	Dowa	2013	0.293886	0.293886	0.293886
153	Dowa	2014	0.266893	0.266893	0.266893
154	Dowa	2015	0.32643	0.32643	0.32643
155	Dowa	2016	0.292995	0.292995	0.292995
156	Dowa	2017	0.188351	0.188351	0.188351
157	Dowa	2018	0.263325	0.263325	0.263325
158	Dowa	2019	0.322082	0.322082	0.322082
159	Dowa	2020	0.305429	0.305429	0.305429
160	Dowa	2021	0.284309	0.284309	0.284309
161	Dowa	2022	0.208752	0.208752	0.208752
162	Karonga	2000	0.511761	0.511761	0.511761
163	Karonga	2001	0.621034	0.621034	0.621034
164	Karonga	2002	0.482836	0.482836	0.482836
165	Karonga	2003	0.362782	0.362782	0.362782
166	Karonga	2004	0.274515	0.274515	0.274515
167	Karonga	2005	0.194637	0.194637	0.194637
168	Karonga	2006	0.175697	0.175697	0.175697
169	Karonga	2007	0.139321	0.139321	0.139321
170	Karonga	2008	0.059583	0.059583	0.059583
171	Karonga	2009	0.031892	0.031892	0.031892
172	Karonga	2010	0.188047	0.188047	0.188047
173	Karonga	2011	0.174992	0.174992	0.174992
174	Karonga	2012	0.158963	0.158963	0.158963
175	Karonga	2013	0.149595	0.149595	0.149595
176	Karonga	2014	0.124794	0.124794	0.124794
177	Karonga	2015	0.10175	0.10175	0.10175
178	Karonga	2016	0.135874	0.135874	0.135874
179	Karonga	2017	0.06152	0.06152	0.06152
180	Karonga	2018	0.099895	0.099895	0.099895
181	Karonga	2019	0.137473	0.137473	0.137473
182	Karonga	2020	0.159092	0.159092	0.159092
183	Karonga	2021	0.172268	0.172268	0.172268
184	Karonga	2022	0.18482	0.18482	0.18482
185	Kasungu	2000	0.405194	0.405194	0.405194
186	Kasungu	2001	0.463439	0.463439	0.463439
187	Kasungu	2002	0.399687	0.399687	0.399687
188	Kasungu	2003	0.3674	0.3674	0.3674
189	Kasungu	2004	0.333177	0.333177	0.333177
190	Kasungu	2005	0.313741	0.313741	0.313741
191	Kasungu	2006	0.331123	0.331123	0.331123
192	Kasungu	2007	0.377951	0.377951	0.377951
193	Kasungu	2008	0.280919	0.280919	0.280919
194	Kasungu	2009	0.226105	0.226105	0.226105
195	Kasungu	2010	0.280654	0.280654	0.280654
196	Kasungu	2011	0.280435	0.280435	0.280435
197	Kasungu	2012	0.302045	0.302045	0.302045
198	Kasungu	2013	0.28895	0.28895	0.28895
199	Kasungu	2014	0.272054	0.272054	0.272054
200	Kasungu	2015	0.241854	0.241854	0.241854
201	Kasungu	2016	0.242869	0.242869	0.242869
202	Kasungu	2017	0.178019	0.178019	0.178019
203	Kasungu	2018	0.225006	0.225006	0.225006
204	Kasungu	2019	0.265319	0.265319	0.265319
205	Kasungu	2020	0.247378	0.247378	0.247378
206	Kasungu	2021	0.221626	0.221626	0.221626
207	Kasungu	2022	0.186623	0.186623	0.186623
208	Likoma	2000	0.331184	0.331184	0.331184
209	Likoma	2001	0.377537	0.377537	0.377537
210	Likoma	2002	0.327442	0.327442	0.327442
211	Likoma	2003	0.292654	0.292654	0.292654
212	Likoma	2004	0.276359	0.276359	0.276359
213	Likoma	2005	0.251862	0.251862	0.251862
214	Likoma	2006	0.24393	0.24393	0.24393
215	Likoma	2007	0.257852	0.257852	0.257852
216	Likoma	2008	0.153156	0.153156	0.153156
217	Likoma	2009	0.103628	0.103628	0.103628
218	Likoma	2010	0.140491	0.140491	0.140491
219	Likoma	2011	0.070701	0.070701	0.070701
220	Likoma	2012	0.095438	0.095438	0.095438
221	Likoma	2013	0.125702	0.125702	0.125702
222	Likoma	2014	0.154284	0.154284	0.154284
223	Likoma	2015	0.158116	0.158116	0.158116
224	Likoma	2016	0.140508	0.140508	0.140508
225	Likoma	2017	0.062951	0.062951	0.062951
226	Likoma	2018	0.123778	0.123778	0.123778
227	Likoma	2019	0.183683	0.183683	0.183683
228	Likoma	2020	0.25701	0.25701	0.25701
229	Likoma	2021	0.339657	0.339657	0.339657
230	Likoma	2022	0.291625	0.291625	0.291625
231	Lilongwe	2000	0.522395	0.522395	0.522395
232	Lilongwe	2001	0.605753	0.605753	0.605753
233	Lilongwe	2002	0.424233	0.424233	0.424233
234	Lilongwe	2003	0.394091	0.394091	0.394091
235	Lilongwe	2004	0.367149	0.367149	0.367149
236	Lilongwe	2005	0.343142	0.343142	0.343142
237	Lilongwe	2006	0.312151	0.312151	0.312151
238	Lilongwe	2007	0.201823	0.201823	0.201823
239	Lilongwe	2008	0.178452	0.178452	0.178452
240	Lilongwe	2009	0.236276	0.236276	0.236276
241	Lilongwe	2010	0.346807	0.346807	0.346807
242	Lilongwe	2011	0.297581	0.297581	0.297581
243	Lilongwe	2012	0.290044	0.290044	0.290044
244	Lilongwe	2013	0.282192	0.282192	0.282192
245	Lilongwe	2014	0.289808	0.289808	0.289808
246	Lilongwe	2015	0.338348	0.338348	0.338348
247	Lilongwe	2016	0.280287	0.280287	0.280287
248	Lilongwe	2017	0.166015	0.166015	0.166015
249	Lilongwe	2018	0.2317	0.2317	0.2317
250	Lilongwe	2019	0.2782	0.2782	0.2782
251	Lilongwe	2020	0.253105	0.253105	0.253105
252	Lilongwe	2021	0.217213	0.217213	0.217213
253	Lilongwe	2022	0.194782	0.194782	0.194782
254	Machinga	2000	0.463685	0.463685	0.463685
255	Machinga	2001	0.611897	0.611897	0.611897
256	Machinga	2002	0.466779	0.466779	0.466779
257	Machinga	2003	0.423524	0.423524	0.423524
258	Machinga	2004	0.359316	0.359316	0.359316
259	Machinga	2005	0.304374	0.304374	0.304374
260	Machinga	2006	0.29875	0.29875	0.29875
261	Machinga	2007	0.187937	0.187937	0.187937
262	Machinga	2008	0.171055	0.171055	0.171055
263	Machinga	2009	0.167837	0.167837	0.167837
264	Machinga	2010	0.31406	0.31406	0.31406
265	Machinga	2011	0.330296	0.330296	0.330296
266	Machinga	2012	0.183526	0.183526	0.183526
267	Machinga	2013	0.214642	0.214642	0.214642
268	Machinga	2014	0.293298	0.293298	0.293298
269	Machinga	2015	0.249819	0.249819	0.249819
270	Machinga	2016	0.330764	0.330764	0.330764
271	Machinga	2017	0.25436	0.25436	0.25436
272	Machinga	2018	0.270756	0.270756	0.270756
273	Machinga	2019	0.281194	0.281194	0.281194
274	Machinga	2020	0.191123	0.191123	0.191123
275	Machinga	2021	0.107353	0.107353	0.107353
276	Machinga	2022	0.137488	0.137488	0.137488
277	Mangochi	2000	0.370842	0.370842	0.370842
278	Mangochi	2001	0.394822	0.394822	0.394822
279	Mangochi	2002	0.325909	0.325909	0.325909
280	Mangochi	2003	0.337306	0.337306	0.337306
281	Mangochi	2004	0.333606	0.333606	0.333606
282	Mangochi	2005	0.330237	0.330237	0.330237
283	Mangochi	2006	0.360542	0.360542	0.360542
284	Mangochi	2007	0.282468	0.282468	0.282468
285	Mangochi	2008	0.23856	0.23856	0.23856
286	Mangochi	2009	0.241719	0.241719	0.241719
287	Mangochi	2010	0.375046	0.375046	0.375046
288	Mangochi	2011	0.307627	0.307627	0.307627
289	Mangochi	2012	0.239541	0.239541	0.239541
290	Mangochi	2013	0.244369	0.244369	0.244369
291	Mangochi	2014	0.270665	0.270665	0.270665
292	Mangochi	2015	0.199572	0.199572	0.199572
293	Mangochi	2016	0.220577	0.220577	0.220577
294	Mangochi	2017	0.244217	0.244217	0.244217
295	Mangochi	2018	0.297772	0.297772	0.297772
296	Mangochi	2019	0.345634	0.345634	0.345634
297	Mangochi	2020	0.325619	0.325619	0.325619
298	Mangochi	2021	0.313614	0.313614	0.313614
299	Mangochi	2022	0.272251	0.272251	0.272251
300	Mchinji	2000	0.456525	0.456525	0.456525
301	Mchinji	2001	0.528061	0.528061	0.528061
302	Mchinji	2002	0.402879	0.402879	0.402879
303	Mchinji	2003	0.347578	0.347578	0.347578
304	Mchinji	2004	0.283112	0.283112	0.283112
305	Mchinji	2005	0.229076	0.229076	0.229076
306	Mchinji	2006	0.251395	0.251395	0.251395
307	Mchinji	2007	0.221091	0.221091	0.221091
308	Mchinji	2008	0.223751	0.223751	0.223751
309	Mchinji	2009	0.253087	0.253087	0.253087
310	Mchinji	2010	0.44969	0.44969	0.44969
311	Mchinji	2011	0.349814	0.349814	0.349814
312	Mchinji	2012	0.29436	0.29436	0.29436
313	Mchinji	2013	0.250026	0.250026	0.250026
314	Mchinji	2014	0.212732	0.212732	0.212732
315	Mchinji	2015	0.177091	0.177091	0.177091
316	Mchinji	2016	0.131225	0.131225	0.131225
317	Mchinji	2017	0.096182	0.096182	0.096182
318	Mchinji	2018	0.184907	0.184907	0.184907
319	Mchinji	2019	0.307124	0.307124	0.307124
320	Mchinji	2020	0.254273	0.254273	0.254273
321	Mchinji	2021	0.198113	0.198113	0.198113
322	Mchinji	2022	0.208022	0.208022	0.208022
323	Mulanje	2000	0.529394	0.529394	0.529394
324	Mulanje	2001	0.70018	0.70018	0.70018
325	Mulanje	2002	0.555396	0.555396	0.555396
326	Mulanje	2003	0.522601	0.522601	0.522601
327	Mulanje	2004	0.400646	0.400646	0.400646
328	Mulanje	2005	0.284216	0.284216	0.284216
329	Mulanje	2006	0.263004	0.263004	0.263004
330	Mulanje	2007	0.139247	0.139247	0.139247
331	Mulanje	2008	0.163875	0.163875	0.163875
332	Mulanje	2009	0.238613	0.238613	0.238613
333	Mulanje	2010	0.182943	0.182943	0.182943
334	Mulanje	2011	0.335455	0.335455	0.335455
335	Mulanje	2012	0.199381	0.199381	0.199381
336	Mulanje	2013	0.180629	0.180629	0.180629
337	Mulanje	2014	0.148925	0.148925	0.148925
338	Mulanje	2015	0.168624	0.168624	0.168624
339	Mulanje	2016	0.293973	0.293973	0.293973
340	Mulanje	2017	0.163025	0.163025	0.163025
341	Mulanje	2018	0.228374	0.228374	0.228374
342	Mulanje	2019	0.274382	0.274382	0.274382
343	Mulanje	2020	0.214642	0.214642	0.214642
344	Mulanje	2021	0.147162	0.147162	0.147162
345	Mulanje	2022	0.119766	0.119766	0.119766
346	Mwanza	2000	0.543574	0.543574	0.543574
347	Mwanza	2001	0.645423	0.645423	0.645423
348	Mwanza	2002	0.525976	0.525976	0.525976
349	Mwanza	2003	0.530802	0.530802	0.530802
350	Mwanza	2004	0.486783	0.486783	0.486783
351	Mwanza	2005	0.457578	0.457578	0.457578
352	Mwanza	2006	0.553516	0.553516	0.553516
353	Mwanza	2007	0.291097	0.291097	0.291097
354	Mwanza	2008	0.131725	0.131725	0.131725
355	Mwanza	2009	0.169612	0.169612	0.169612
356	Mwanza	2010	0.28965	0.28965	0.28965
357	Mwanza	2011	0.316122	0.316122	0.316122
358	Mwanza	2012	0.221929	0.221929	0.221929
359	Mwanza	2013	0.257877	0.257877	0.257877
360	Mwanza	2014	0.275277	0.275277	0.275277
361	Mwanza	2015	0.170084	0.170084	0.170084
362	Mwanza	2016	0.213555	0.213555	0.213555
363	Mwanza	2017	0.111706	0.111706	0.111706
364	Mwanza	2018	0.229526	0.229526	0.229526
365	Mwanza	2019	0.38103	0.38103	0.38103
366	Mwanza	2020	0.30488	0.30488	0.30488
367	Mwanza	2021	0.227722	0.227722	0.227722
368	Mwanza	2022	0.309037	0.309037	0.309037
369	Mzimba	2000	0.363134	0.363134	0.363134
370	Mzimba	2001	0.415995	0.415995	0.415995
371	Mzimba	2002	0.350549	0.350549	0.350549
372	Mzimba	2003	0.308733	0.308733	0.308733
373	Mzimba	2004	0.263629	0.263629	0.263629
374	Mzimba	2005	0.231963	0.231963	0.231963
375	Mzimba	2006	0.249282	0.249282	0.249282
376	Mzimba	2007	0.297028	0.297028	0.297028
377	Mzimba	2008	0.179853	0.179853	0.179853
378	Mzimba	2009	0.134896	0.134896	0.134896
379	Mzimba	2010	0.165407	0.165407	0.165407
380	Mzimba	2011	0.148731	0.148731	0.148731
381	Mzimba	2012	0.141297	0.141297	0.141297
382	Mzimba	2013	0.168081	0.168081	0.168081
383	Mzimba	2014	0.182006	0.182006	0.182006
384	Mzimba	2015	0.127176	0.127176	0.127176
385	Mzimba	2016	0.126877	0.126877	0.126877
386	Mzimba	2017	0.074048	0.074048	0.074048
387	Mzimba	2018	0.106727	0.106727	0.106727
388	Mzimba	2019	0.132881	0.132881	0.132881
389	Mzimba	2020	0.141573	0.141573	0.141573
390	Mzimba	2021	0.142009	0.142009	0.142009
391	Mzimba	2022	0.151135	0.151135	0.151135
392	Neno	2000	0.479514	0.479514	0.479514
393	Neno	2001	0.583133	0.583133	0.583133
394	Neno	2002	0.476269	0.476269	0.476269
395	Neno	2003	0.499932	0.499932	0.499932
396	Neno	2004	0.474845	0.474845	0.474845
397	Neno	2005	0.462595	0.462595	0.462595
398	Neno	2006	0.584955	0.584955	0.584955
399	Neno	2007	0.301348	0.301348	0.301348
400	Neno	2008	0.174185	0.174185	0.174185
401	Neno	2009	0.187901	0.187901	0.187901
402	Neno	2010	0.378836	0.378836	0.378836
403	Neno	2011	0.351343	0.351343	0.351343
404	Neno	2012	0.199524	0.199524	0.199524
405	Neno	2013	0.200591	0.200591	0.200591
406	Neno	2014	0.202291	0.202291	0.202291
407	Neno	2015	0.158065	0.158065	0.158065
408	Neno	2016	0.177741	0.177741	0.177741
409	Neno	2017	0.095166	0.095166	0.095166
410	Neno	2018	0.213447	0.213447	0.213447
411	Neno	2019	0.369219	0.369219	0.369219
412	Neno	2020	0.267989	0.267989	0.267989
413	Neno	2021	0.169875	0.169875	0.169875
414	Neno	2022	0.226223	0.226223	0.226223
415	Nkhata Bay	2000	0.390735	0.390735	0.390735
416	Nkhata Bay	2001	0.456789	0.456789	0.456789
417	Nkhata Bay	2002	0.38764	0.38764	0.38764
418	Nkhata Bay	2003	0.336	0.336	0.336
419	Nkhata Bay	2004	0.29144	0.29144	0.29144
420	Nkhata Bay	2005	0.251932	0.251932	0.251932
421	Nkhata Bay	2006	0.282561	0.282561	0.282561
422	Nkhata Bay	2007	0.300249	0.300249	0.300249
423	Nkhata Bay	2008	0.188573	0.188573	0.188573
424	Nkhata Bay	2009	0.142465	0.142465	0.142465
425	Nkhata Bay	2010	0.256177	0.256177	0.256177
426	Nkhata Bay	2011	0.193917	0.193917	0.193917
427	Nkhata Bay	2012	0.1742	0.1742	0.1742
428	Nkhata Bay	2013	0.211937	0.211937	0.211937
429	Nkhata Bay	2014	0.238871	0.238871	0.238871
430	Nkhata Bay	2015	0.272361	0.272361	0.272361
431	Nkhata Bay	2016	0.304518	0.304518	0.304518
432	Nkhata Bay	2017	0.141792	0.141792	0.141792
433	Nkhata Bay	2018	0.164584	0.164584	0.164584
434	Nkhata Bay	2019	0.182488	0.182488	0.182488
435	Nkhata Bay	2020	0.176599	0.176599	0.176599
436	Nkhata Bay	2021	0.154364	0.154364	0.154364
437	Nkhata Bay	2022	0.168812	0.168812	0.168812
438	Nkhotakota	2000	0.543068	0.543068	0.543068
439	Nkhotakota	2001	0.621261	0.621261	0.621261
440	Nkhotakota	2002	0.571402	0.571402	0.571402
441	Nkhotakota	2003	0.556379	0.556379	0.556379
442	Nkhotakota	2004	0.555728	0.555728	0.555728
443	Nkhotakota	2005	0.578333	0.578333	0.578333
444	Nkhotakota	2006	0.636758	0.636758	0.636758
445	Nkhotakota	2007	0.743443	0.743443	0.743443
446	Nkhotakota	2008	0.508999	0.508999	0.508999
447	Nkhotakota	2009	0.335302	0.335302	0.335302
448	Nkhotakota	2010	0.425579	0.425579	0.425579
449	Nkhotakota	2011	0.326901	0.326901	0.326901
450	Nkhotakota	2012	0.265737	0.265737	0.265737
451	Nkhotakota	2013	0.321885	0.321885	0.321885
452	Nkhotakota	2014	0.36625	0.36625	0.36625
453	Nkhotakota	2015	0.386856	0.386856	0.386856
454	Nkhotakota	2016	0.378737	0.378737	0.378737
455	Nkhotakota	2017	0.265703	0.265703	0.265703
456	Nkhotakota	2018	0.316547	0.316547	0.316547
457	Nkhotakota	2019	0.353951	0.353951	0.353951
458	Nkhotakota	2020	0.343956	0.343956	0.343956
459	Nkhotakota	2021	0.346996	0.346996	0.346996
460	Nkhotakota	2022	0.265698	0.265698	0.265698
461	Nsanje	2000	0.29407	0.29407	0.29407
462	Nsanje	2001	0.34423	0.34423	0.34423
463	Nsanje	2002	0.279131	0.279131	0.279131
464	Nsanje	2003	0.257955	0.257955	0.257955
465	Nsanje	2004	0.140765	0.140765	0.140765
466	Nsanje	2005	0.067631	0.067631	0.067631
467	Nsanje	2006	0.06432	0.06432	0.06432
468	Nsanje	2007	0.07787	0.07787	0.07787
469	Nsanje	2008	0.097248	0.097248	0.097248
470	Nsanje	2009	0.132024	0.132024	0.132024
471	Nsanje	2010	0.146268	0.146268	0.146268
472	Nsanje	2011	0.105374	0.105374	0.105374
473	Nsanje	2012	0.048199	0.048199	0.048199
474	Nsanje	2013	0.089644	0.089644	0.089644
475	Nsanje	2014	0.113321	0.113321	0.113321
476	Nsanje	2015	0.089195	0.089195	0.089195
477	Nsanje	2016	0.077474	0.077474	0.077474
478	Nsanje	2017	0.06498	0.06498	0.06498
479	Nsanje	2018	0.148612	0.148612	0.148612
480	Nsanje	2019	0.27392	0.27392	0.27392
481	Nsanje	2020	0.168121	0.168121	0.168121
482	Nsanje	2021	0.089939	0.089939	0.089939
483	Nsanje	2022	0.081846	0.081846	0.081846
484	Ntcheu	2000	0.294489	0.294489	0.294489
485	Ntcheu	2001	0.279652	0.279652	0.279652
486	Ntcheu	2002	0.217044	0.217044	0.217044
487	Ntcheu	2003	0.251153	0.251153	0.251153
488	Ntcheu	2004	0.25561	0.25561	0.25561
489	Ntcheu	2005	0.261859	0.261859	0.261859
490	Ntcheu	2006	0.348311	0.348311	0.348311
491	Ntcheu	2007	0.188614	0.188614	0.188614
492	Ntcheu	2008	0.134243	0.134243	0.134243
493	Ntcheu	2009	0.139974	0.139974	0.139974
494	Ntcheu	2010	0.291363	0.291363	0.291363
495	Ntcheu	2011	0.193289	0.193289	0.193289
496	Ntcheu	2012	0.1061	0.1061	0.1061
497	Ntcheu	2013	0.14182	0.14182	0.14182
498	Ntcheu	2014	0.199514	0.199514	0.199514
499	Ntcheu	2015	0.136693	0.136693	0.136693
500	Ntcheu	2016	0.104397	0.104397	0.104397
501	Ntcheu	2017	0.11103	0.11103	0.11103
502	Ntcheu	2018	0.232054	0.232054	0.232054
503	Ntcheu	2019	0.39076	0.39076	0.39076
504	Ntcheu	2020	0.305898	0.305898	0.305898
505	Ntcheu	2021	0.237849	0.237849	0.237849
506	Ntcheu	2022	0.129335	0.129335	0.129335
507	Ntchisi	2000	0.493582	0.493582	0.493582
508	Ntchisi	2001	0.561154	0.561154	0.561154
509	Ntchisi	2002	0.495021	0.495021	0.495021
510	Ntchisi	2003	0.491544	0.491544	0.491544
511	Ntchisi	2004	0.495733	0.495733	0.495733
512	Ntchisi	2005	0.518403	0.518403	0.518403
513	Ntchisi	2006	0.565849	0.565849	0.565849
514	Ntchisi	2007	0.671681	0.671681	0.671681
515	Ntchisi	2008	0.468287	0.468287	0.468287
516	Ntchisi	2009	0.357869	0.357869	0.357869
517	Ntchisi	2010	0.362282	0.362282	0.362282
518	Ntchisi	2011	0.332181	0.332181	0.332181
519	Ntchisi	2012	0.33464	0.33464	0.33464
520	Ntchisi	2013	0.331198	0.331198	0.331198
521	Ntchisi	2014	0.317568	0.317568	0.317568
522	Ntchisi	2015	0.413858	0.413858	0.413858
523	Ntchisi	2016	0.362004	0.362004	0.362004
524	Ntchisi	2017	0.216041	0.216041	0.216041
525	Ntchisi	2018	0.276312	0.276312	0.276312
526	Ntchisi	2019	0.315893	0.315893	0.315893
527	Ntchisi	2020	0.32407	0.32407	0.32407
528	Ntchisi	2021	0.331188	0.331188	0.331188
529	Ntchisi	2022	0.1924	0.1924	0.1924
530	Phalombe	2000	0.493491	0.493491	0.493491
531	Phalombe	2001	0.662697	0.662697	0.662697
532	Phalombe	2002	0.512749	0.512749	0.512749
533	Phalombe	2003	0.464661	0.464661	0.464661
534	Phalombe	2004	0.365641	0.365641	0.365641
535	Phalombe	2005	0.253857	0.253857	0.253857
536	Phalombe	2006	0.227657	0.227657	0.227657
537	Phalombe	2007	0.118761	0.118761	0.118761
538	Phalombe	2008	0.153129	0.153129	0.153129
539	Phalombe	2009	0.345325	0.345325	0.345325
540	Phalombe	2010	0.183645	0.183645	0.183645
541	Phalombe	2011	0.349079	0.349079	0.349079
542	Phalombe	2012	0.187439	0.187439	0.187439
543	Phalombe	2013	0.159923	0.159923	0.159923
544	Phalombe	2014	0.138873	0.138873	0.138873
545	Phalombe	2015	0.15037	0.15037	0.15037
546	Phalombe	2016	0.287803	0.287803	0.287803
547	Phalombe	2017	0.176051	0.176051	0.176051
548	Phalombe	2018	0.221686	0.221686	0.221686
549	Phalombe	2019	0.252701	0.252701	0.252701
550	Phalombe	2020	0.180173	0.180173	0.180173
551	Phalombe	2021	0.109196	0.109196	0.109196
552	Phalombe	2022	0.079751	0.079751	0.079751
553	Rumphi	2000	0.386533	0.386533	0.386533
554	Rumphi	2001	0.469267	0.469267	0.469267
555	Rumphi	2002	0.342706	0.342706	0.342706
556	Rumphi	2003	0.249614	0.249614	0.249614
557	Rumphi	2004	0.167328	0.167328	0.167328
558	Rumphi	2005	0.096682	0.096682	0.096682
559	Rumphi	2006	0.111103	0.111103	0.111103
560	Rumphi	2007	0.138695	0.138695	0.138695
561	Rumphi	2008	0.042173	0.042173	0.042173
562	Rumphi	2009	0.033604	0.033604	0.033604
563	Rumphi	2010	0.116186	0.116186	0.116186
564	Rumphi	2011	0.095456	0.095456	0.095456
565	Rumphi	2012	0.077299	0.077299	0.077299
566	Rumphi	2013	0.101355	0.101355	0.101355
567	Rumphi	2014	0.109505	0.109505	0.109505
568	Rumphi	2015	0.099809	0.099809	0.099809
569	Rumphi	2016	0.092702	0.092702	0.092702
570	Rumphi	2017	0.047721	0.047721	0.047721
571	Rumphi	2018	0.092002	0.092002	0.092002
572	Rumphi	2019	0.147925	0.147925	0.147925
573	Rumphi	2020	0.202217	0.202217	0.202217
574	Rumphi	2021	0.267299	0.267299	0.267299
575	Rumphi	2022	0.248101	0.248101	0.248101
576	Salima	2000	0.545842	0.545842	0.545842
577	Salima	2001	0.619902	0.619902	0.619902
578	Salima	2002	0.530408	0.530408	0.530408
579	Salima	2003	0.529729	0.529729	0.529729
580	Salima	2004	0.526327	0.526327	0.526327
581	Salima	2005	0.552961	0.552961	0.552961
582	Salima	2006	0.573444	0.573444	0.573444
583	Salima	2007	0.551217	0.551217	0.551217
584	Salima	2008	0.410843	0.410843	0.410843
585	Salima	2009	0.378084	0.378084	0.378084
586	Salima	2010	0.46229	0.46229	0.46229
587	Salima	2011	0.333737	0.333737	0.333737
588	Salima	2012	0.248171	0.248171	0.248171
589	Salima	2013	0.2611	0.2611	0.2611
590	Salima	2014	0.264888	0.264888	0.264888
591	Salima	2015	0.328344	0.328344	0.328344
592	Salima	2016	0.301914	0.301914	0.301914
593	Salima	2017	0.202588	0.202588	0.202588
594	Salima	2018	0.294576	0.294576	0.294576
595	Salima	2019	0.382157	0.382157	0.382157
596	Salima	2020	0.324607	0.324607	0.324607
597	Salima	2021	0.295222	0.295222	0.295222
598	Salima	2022	0.213722	0.213722	0.213722
599	Thyolo	2000	0.43121	0.43121	0.43121
600	Thyolo	2001	0.563531	0.563531	0.563531
601	Thyolo	2002	0.418377	0.418377	0.418377
602	Thyolo	2003	0.393032	0.393032	0.393032
603	Thyolo	2004	0.243063	0.243063	0.243063
604	Thyolo	2005	0.118853	0.118853	0.118853
605	Thyolo	2006	0.097281	0.097281	0.097281
606	Thyolo	2007	0.075685	0.075685	0.075685
607	Thyolo	2008	0.085435	0.085435	0.085435
608	Thyolo	2009	0.117249	0.117249	0.117249
609	Thyolo	2010	0.184531	0.184531	0.184531
610	Thyolo	2011	0.155484	0.155484	0.155484
611	Thyolo	2012	0.041352	0.041352	0.041352
612	Thyolo	2013	0.060895	0.060895	0.060895
613	Thyolo	2014	0.073384	0.073384	0.073384
614	Thyolo	2015	0.074938	0.074938	0.074938
615	Thyolo	2016	0.108872	0.108872	0.108872
616	Thyolo	2017	0.063658	0.063658	0.063658
617	Thyolo	2018	0.138561	0.138561	0.138561
618	Thyolo	2019	0.228473	0.228473	0.228473
619	Thyolo	2020	0.195225	0.195225	0.195225
620	Thyolo	2021	0.147968	0.147968	0.147968
621	Thyolo	2022	0.159824	0.159824	0.159824
622	Zomba	2000	0.40517	0.40517	0.40517
623	Zomba	2001	0.609545	0.609545	0.609545
624	Zomba	2002	0.438367	0.438367	0.438367
625	Zomba	2003	0.40731	0.40731	0.40731
626	Zomba	2004	0.319961	0.319961	0.319961
627	Zomba	2005	0.225269	0.225269	0.225269
628	Zomba	2006	0.218366	0.218366	0.218366
629	Zomba	2007	0.100714	0.100714	0.100714
630	Zomba	2008	0.122283	0.122283	0.122283
631	Zomba	2009	0.156525	0.156525	0.156525
632	Zomba	2010	0.187532	0.187532	0.187532
633	Zomba	2011	0.391623	0.391623	0.391623
634	Zomba	2012	0.176082	0.176082	0.176082
635	Zomba	2013	0.12538	0.12538	0.12538
636	Zomba	2014	0.12684	0.12684	0.12684
637	Zomba	2015	0.171951	0.171951	0.171951
638	Zomba	2016	0.321081	0.321081	0.321081
639	Zomba	2017	0.20556	0.20556	0.20556
640	Zomba	2018	0.275066	0.275066	0.275066
641	Zomba	2019	0.338558	0.338558	0.338558
642	Zomba	2020	0.22258	0.22258	0.22258
643	Zomba	2021	0.11991	0.11991	0.11991
644	Zomba	2022	0.145307	0.145307	0.145307

### Model validation

**Figure 7.**
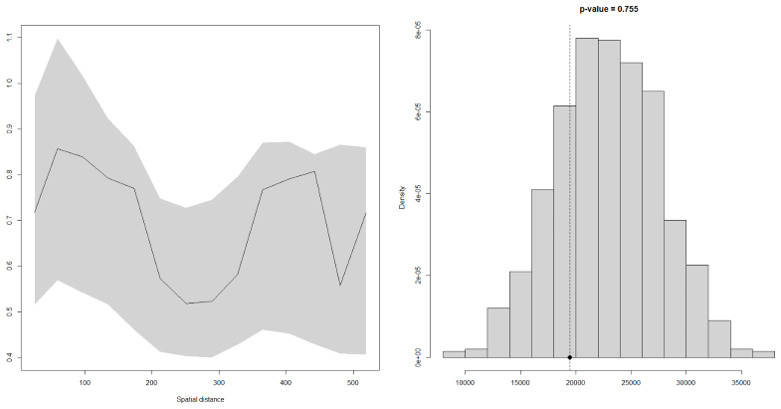
Validity of the assumed covariance model for the spatial correlation. The empirical semi-variogram (solid line) falls within the 95% tolerance intervals (dashed lines), indicating that the adopted covariance model was compatible with the data.

Using variogram based techniques described above, the model above was tested for evidence for spatial corelation. The results of this process are shown in the Figure 7 below. Since the empirical semi-variogram (solid line) falls within the 95% confidence intervals (grey envelope), then this shows that the model is valid; the model for malaria prevalence is therefore compatible with the data.

## Discussion

The past two decades have been characterised by a substantial scale-up of available malaria control tools in Malawi. Historically, the country has been known to be ahead of many other African countries with regard to its malaria policies ^23^ and is usually among the first to respond to new or available malaria interventions. For example, in 1993, the country was the first in Africa to change its first-line therapy for uncomplicated malaria from chloroquine to sulfadoxine-pyrimethamine (S.P.) ^24^. With increasing evidence of a reduced cure rate of S.P., to as low as 82%, the country was again first to change the first-line treatment for uncomplicated malaria from S.P. to artemether-lumefantrine (A.L.) in 2007 for all adults and children over the age of 5 who test positive for malaria using a rapid diagnostic test ^23^. Within this period between 2000–2022, there have been four strategic malaria plans that have been implemented ^20^. Under these strategies, several key things have happened including the introduction of rapid diagnostic tests that scaled up the testing and treatment of malaria, introduction of artemisinin combination therapies as the first line treatment for uncomplicated malaria, use of indoor residual spraying, mass net distribution of long-lasting insecticide treated nets including PBO nets that were introduced in 2018 and more recently, the roll out of the malaria vaccine. Indeed, the last two decades have been an evolving period for malaria control. Based on what we know and validated by the present analysis, malaria transmission is decreasing and becoming more heterogeneous at subnational levels. There is a need for more robust tools to guide targeted control efforts in the remaining hotspot areas.

The current Malawi malaria strategic plan that started in 2016 ended in December 2022. The strategy aimed to reduce malaria incidence by at least 50% from a 2016 baseline of 386 per 1000 population to 193 per 1000 and reduce malaria deaths by at least 50% from 23 per 100,000 population to 12 per 100,000 population by 2022. Based on the 2019 malaria programme review, the NMCP are on track with their indicators. The subsequent 2023 to 2030 malaria strategic plan is crucial to the elimination of malaria. To directly inform the next strategic plan, we analysed the last 20 years of available prevalence data to understand the changing transmission patterns in the 21-year period between 2000 and 2021. Specifically, we aimed to map the malaria prevalence estimates and their associated uncertainty for the whole surface of Malawi at higher resolution (1x1 Km grids) for the period of interest and to map the malaria prevalence estimates and their associated uncertainty at district level for the period of interest.

From the analysis, within this 22-year period, we have demonstrated that malaria transmission in Malawi is becoming more heterogeneous. There are hotspots of high transmission and areas of very low transmission. This is due to varied climatic conditions, vector and parasite resistance, conducive environmental factors in urban and rural areas and, varied intervention uptake in the different parts of the country. The years between 2000 and 2010 were associated with minimal malaria funding available to the country ^25^, which is evidenced by the high prevalence of malaria in 2006, based on this present analysis. In the follow up years, between 2010 and 2015, massive donor investments and local support for malaria control were made available leading to an increase in the coverage of interventions and a decline in prevalence ^26^. In the absence of known climate anomalies that could have led to a sudden decline in malaria infection during this period, it can be interpolated that the decline that was observed in this period was due to the expansion of malaria control initiatives in the country ^17^.

Understanding and predicting patterns of transmission risk forms an essential component of an effective elimination campaign, allowing limited resources for control and elimination to be targeted cost-effectively. Cognizant of this, WHO recently updated its guidance to view malaria transmission as a continuum within countries, encouraging countries to use surveillance as one of the core interventions and to incorporate malaria early warning systems that can predict outbreaks or unexpected and short-term disease changes to effectively allocate resources ^13^.

In the present analysis, we use Temperature Suitability Index (TSI) for malaria. This measure has been used in various fields, including ecology, agriculture, and public health, to estimate the suitability of environmental conditions, in this case temperature, for a specific organism, crop, or disease vector. In the context of malaria, TSI is an indicator of how suitable the ambient temperature is for the transmission and development of the malaria parasite within its mosquito vector. Temperature plays a crucial role in the life cycle and transmission dynamics of malaria parasites and their mosquito vectors. The development rate of the parasite within the mosquito (sporogonic cycle) and the mosquito's life span are both temperature-dependent. There is an optimal temperature range that allows the malaria parasite to complete its development within the mosquito, and for the mosquito to survive long enough to transmit the parasite to a new host.The TSI for malaria transmission takes into account these temperature-dependent relationships and can be used as a covariate in models to predict the spatial and temporal distribution of malaria risk. By incorporating TSI into a model, researchers can better understand how environmental factors, such as temperature, contribute to the observed patterns of malaria transmission and prevalence.

A key strength in the current analysis is that we have leveraged data from multiple surveys, which in turn improves the predictions of prevalence, as opposed to using single survey data which often time may contain data that is sparse for high resolution predictions. So far, in Malawi, efforts to model spatio-temporal heterogeneity in malaria have focused on parasite prevalence (infection) data from household surveys, because of concerns about the quality and completeness of routine clinical malaria case data from the national District Health Information System (DHIS2). National malaria prevalence surveys, however, are costly and are only conducted every 2–4 years and, while parasite prevalence reflects transmission, it does not necessarily align with the disease burden in higher transmission settings. The funding landscape is also becoming increasingly unpredictable. The 2019 Malawi malaria indicator household survey was not conducted due to funding constraints and COVID-19 delays, interfering with national progress tracking. Keen to maximise the use of routine data, the NMCP has made substantial investments in the DHIS2 data since 2016, resulting in steady data quality improvements in terms of timeliness, completion rates and data accuracy of routine data. The 2019 WHO Malawi mid-term review confirmed this achievement that routine case data can be confidently used for surveillance and decision-making. Future descriptions of subnational malaria risk may have to utilise routinely collected data and consider using composite measures. Employing these models in a situation of reliable routinely collected data may be less ideal. Despite this, to date this is the most detailed/ up to date description of malaria prevalence for the last two decades in Malawi.

## Conclusions & recommendations

Malaria remains a public health concern, especially in Malawi. The last two decades have been characterized by a scale-up of available control tools leading to a reduction in prevalence. Decreasing malaria transmission has however contributed to "heterogeneous" transmission landscapes in different parts of countries. The prevalence estimates from the modelling need to be triangulated with routinely collected data. Efforts to control malaria beyond 2022 should focus on targeting of control measures in areas of highest need.

## Consent

Secondary aggregate survey data have been used from 2000. The authors have assumed all original sampling have been cleared by the relevant institutional review boards in Malawi

## Data Availability

Figshare. Malaria Prevalence Survey data Malawi 2000–2022_final.csv. DOI: https://doi.org/10.6084/m9.figshare.22587580.v1. The dataset includes information on survey cluster locations, individual test results for Plasmodium falciparum, sampled individuals' ages, and temperature suitability index values for each location and time point. The analysis was done in the R statistical software environment using the PrevMap package [Giorgi & Diggle 2017] which are both open-source. Data are available under the terms of the Creative Commons Attribution 4.0 International license (CC-BY 4.0).
