# Supplementary material for: Two decades of malaria control in Malawi: Geostatistical Analysis of the changing malaria prevalence from 2000-2022
Source: Wellcome Open Res. 2024 Jan 8;8:264. Originally published 2023 Jun 21. [Version 2] doi: 10.12688/wellcomeopenres.19390.2 (PMC11097645; doi:10.12688/wellcomeopenres.19390.2)
Supplement: Peer review history

# Reviewer report: Reviewer response for version 2

**DOI:** 10.21956/wellcomeopenres.23017.r72387

**Published:** 2024-02-27

**Authors:** Manuela Runge

**Affiliations:** 1 Northwestern University, Evanston, Illinois, USA

The reviewer thanks the authors for their diligent replies and edits on the manuscript.

 The authors adequately address the concerns highlighted, encompassing updated references, cross-references to earlier work from the authors, revision of previously unclear sentences, edited discussion to better connect with the introduction, and correction of typos.

 Some inquiries were out of scope, such as district-level timelines and sub-district estimates are expected for future work. Overall, version 2 is a much-improved manuscript.

 A few minor format issues could be considered in a  possible revision:

 - PfPR 2–10   vs   PfPR2_10, both appear in the text

 - The sentence on exceedance probabilities that were not calculated could be removed as nowhere else mentioned after the revision.

 - Temperature Suitability Index (TSI) abbreviation was reintroduced in the discussion, although TSI has already been defined earlier.

Is the work clearly and accurately presented and does it cite the current literature?

Partly

If applicable, is the statistical analysis and its interpretation appropriate?

I cannot comment. A qualified statistician is required.

Are all the source data underlying the results available to ensure full reproducibility?

Partly

Is the study design appropriate and is the work technically sound?

Yes

Are the conclusions drawn adequately supported by the results?

Partly

Are sufficient details of methods and analysis provided to allow replication by others?

Partly

Reviewer Expertise:

Malaria epidemiologist and mathematical modeller of malaria intervention impact.

I confirm that I have read this submission and believe that I have an appropriate level of expertise to confirm that it is of an acceptable scientific standard.

# Reviewer report: Reviewer response for version 2

**DOI:** 10.21956/wellcomeopenres.23017.r72386

**Published:** 2024-02-22

**Authors:** Ebenezer Krampah Aidoo

**Affiliations:** 1 Accra Technical University, Accra, Ghana

The period of the study isn't clear.

**"To directly inform the next strategic plan, we analyzed the last 20 years of available prevalence data to understand the changing transmission patterns in the 21 years between 2000 and 2021"**

**"From the analysis, within these 22 years....**.,

 Clarity is needed on this and must be reflected in the title whichever is applicable.

 “ *For a study that analyzed the last 20 years of available prevalence data to understand the changing transmission patterns in the 21-year period between 2000 and 2021*”, it would have been ideal to support the study with some years (not necessarily 20 years) of entomological data. **The absence of entomological data limits the discussion about abundant vectors in Malawi and whether their relative distribution has changed before and after the implementation of malaria controls. Consider this as a limitation of your study**

Is the work clearly and accurately presented and does it cite the current literature?

Yes

If applicable, is the statistical analysis and its interpretation appropriate?

I cannot comment. A qualified statistician is required.

Are all the source data underlying the results available to ensure full reproducibility?

Yes

Is the study design appropriate and is the work technically sound?

Yes

Are the conclusions drawn adequately supported by the results?

Yes

Are sufficient details of methods and analysis provided to allow replication by others?

Yes

Reviewer Expertise:

My research interests are in the areas of malaria epidemiology, host-parasite interactions, dynamics of disease transmission, antimalarial drug resistance and use of molecular tools to better understand the transmission of malaria, other infectious diseases (COVID-19, Helicobacter pylori infection etc) and neglected tropical diseases (Schistosomiasis, Soil-transmitted helminth infection etc

I confirm that I have read this submission and believe that I have an appropriate level of expertise to confirm that it is of an acceptable scientific standard.

# Reviewer report: Reviewer response for version 2

**DOI:** 10.21956/wellcomeopenres.23017.r72388

**Published:** 2024-01-31

**Authors:** Adilson DePINA

**Affiliations:** 1 Malaria Elimination Program, CCS-SIDA / Ministry of Health, Praia, Santiago, Cape Verde; 2 Laboratório de Entomologia Médica, Instituto Nacional de Saúde Pública, Praia, Santiago, Cape Verde

Great work done by the authors, improving the manuscript quality.

Is the work clearly and accurately presented and does it cite the current literature?

Partly

If applicable, is the statistical analysis and its interpretation appropriate?

I cannot comment. A qualified statistician is required.

Are all the source data underlying the results available to ensure full reproducibility?

Yes

Is the study design appropriate and is the work technically sound?

Yes

Are the conclusions drawn adequately supported by the results?

Yes

Are sufficient details of methods and analysis provided to allow replication by others?

Partly

Reviewer Expertise:

Public Health, Malaria Elimination, Vector control, Program Management, Malaria Epidemiology

I confirm that I have read this submission and believe that I have an appropriate level of expertise to confirm that it is of an acceptable scientific standard.

# Reviewer report: Reviewer response for version 1

**DOI:** 10.21956/wellcomeopenres.21477.r63178

**Published:** 2023-08-18

**Authors:** Mark M Janko

**Affiliations:** 1 Duke University, Durham, North Carolina, USA

The authors set out to describe the spatio-temporal patterns of malaria prevalence from 2000-2022 using a compiled data set of parasite surveys spanning this same period. The authors do a nice job of summarizing the advances in malaria control, as well as challenges. I have a number of comments that would improve the manuscript. 

1. This paper builds on a previous analysis that spans 2010-2017. However, the model needs to be described better. The current description (including in the previous analysis) is incomplete. For example, the authors note that they adopt an exponential correlation function to describe spatial and temporal correlation. They further note that they used empirical variogram based methods to test for spatial autocorrelation. What about the temporal correlation structure? 
2. The description of the variogram-based approach does not make sense. The authors note that they generated these variograms under their model to construct a 95% confidence set. By construction the parameters identified by the model would fall within the 95% confidence limits of variograms generated under the same model. But the authors then note that evidence of spatial autocorrelation would be present if the empirical variogram obtained from the data fell outside the confidence set. In the model validation section, they then state that their model is compatible with the data because the empirical variogram is within the 95% confidence limits. The presentation of this is unclear to this reviewer.
Recommendation: Revise the discussion over model validation and specification of the covariance parameters. For model validation, it would be better to compare models using out-of-sample cross validation. 
3. The notation is unclear. Specifically, the authors are inconsistent with how space and time are denoted. For example, Y_i is a malaria case count at location x_i and time t_i. But in the subsequent equations there is no subscript i, just x and t. 
4. There is no discussion of how Z(x,t) are modeled. Rather, it is just stated that they are modeled as random effects? How are these parameterized? This is important since, for any given space-time location, there are two random effects being estimated—S(x,t) and Z(x,t)—meaning there are potential identifiability issues since two parameters are being estimated for each row of data.
Recommendation: Fully describe the model. 
5. The authors state that they are interested in PFPR in 2-10 year olds. However, the model only includes min age and max age as covariates. It is not at all clear how these results are then translated to a prevalence surface of 2-10 year olds. More generally, there are not really any descriptive statistics for any of the covariates in the model, nor are there parameter estimates. In the attached dataset for the paper, minimum and maximum (LoAge and UpAge) seem to correspond to mA and MA, but this is not clear. Further, age is not recorded in whole years, which suggests that some kind of average is being computed.
Recommendation: Refit the model with 5-year age groups modeled as either a random effect or as indicator variables. This will require redefining the outcome as counts in locations during years among different ages. However, this will actually provide much more information for policy-makers and make an important contribution to understanding the distribution of malaria across Malawi and how it varies demographically. To construct a covariate for the spatial prediction component, 5-year age data are available from WorldPop. Additionally, the authors should add a table of descriptive statistics for the covariates and outcome data. 
6. The authors note a number of advances (and challenges to malaria control). Why not include these as indicator variables in the model. For example, an indicator could be included in years 2018-2022 to indicate that PBO-based bed nets were introduced. Similarly, the time period spanning the covid pandemic could also be included as an indicator variable. Right now, all of the variability in prevalence that is due to those factors is absorbed in the random effects.
Recommendation: Refit the model to account for (in some way, even if basic) the advances/setbacks in malaria control. 
7. The references are not entirely appropriate. For example, the authors cite a paper on malaria chemotherapy from 2010 to indicate that malaria is a public health problem. Why not the WHO world malaria report that is released every year? Also, the paper is about *P. falciparum *malaria, but they cite a paper on *P. knowlesi*.
Recommendation: The authors need to revisit the references and update them to be the most relevant citations. 

Is the work clearly and accurately presented and does it cite the current literature?

No

If applicable, is the statistical analysis and its interpretation appropriate?

Partly

Are all the source data underlying the results available to ensure full reproducibility?

Partly

Is the study design appropriate and is the work technically sound?

Partly

Are the conclusions drawn adequately supported by the results?

Partly

Are sufficient details of methods and analysis provided to allow replication by others?

No

Reviewer Expertise:

Hierarchical Bayesian spatial and spatio-temporal modeling; Malaria epidemiology

I confirm that I have read this submission and believe that I have an appropriate level of expertise to confirm that it is of an acceptable scientific standard, however I have significant reservations, as outlined above.

# Response

**Published:** 2023-12-22

**Authors:** Donnie Mategula

**Affiliations:** Malawi-Liverpool Wellcome Trust Clinical Research Programme, Malawi

**Dear Mark M Janko, Many thanks for your helpful and thoughtful comments. We have re-submitted an updated manuscript that addresses your suggestions and concerns. Please find our detailed responses below.**

 The authors set out to describe the spatio-temporal patterns of malaria prevalence from 2000-2022 using a compiled data set of parasite surveys spanning this same period. The authors do a nice job of summarizing the advances in malaria control, as well as challenges. I have a number of comments that would improve the manuscript. This paper builds on a previous analysis that spans 2010-2017. However, the model needs to be described better. The current description (including in the previous analysis) is incomplete. For example, the authors note that they adopt an exponential correlation function to describe spatial and temporal correlation. They further note that they used empirical variogram based methods to test for spatial autocorrelation. What about the temporal correlation structure?   The description of the variogram-based approach does not make sense. The authors note that they generated these variograms under their model to construct a 95% confidence set. By construction the parameters identified by the model would fall within the 95% confidence limits of variograms generated under the same model. But the authors then note that evidence of spatial autocorrelation would be present if the empirical variogram obtained from the data fell outside the confidence set. In the model validation section, they then state that their model is compatible with the data because the empirical variogram is within the 95% confidence limits. The presentation of this is unclear to this reviewer. Recommendation: Revise the discussion over model validation and specification of the covariance parameters. For model validation, it would be better to compare models using out-of-sample cross validation. The notation is unclear. Specifically, the authors are inconsistent with how space and time are denoted. For example, Y_i is a malaria case count at location x_i and time t_i. But in the subsequent equations there is no subscript i, just x and t. There is no discussion of how Z(x,t) are modeled. Rather, it is just stated that they are modeled as random effects? How are these parameterized? This is important since, for any given space-time location, there are two random effects being estimated—S(x,t) and Z(x,t)—meaning there are potential identifiability issues since two parameters are being estimated for each row of data. Recommendation: Fully describe the model. **Thank you very much. We have added a reference that fully describes the modeling approach.**

 The authors state that they are interested in PFPR in 2-10 year olds. However, the model only includes min age and max age as covariates. It is not at all clear how these results are then translated to a prevalence surface of 2-10 year olds. More generally, there are not really any descriptive statistics for any of the covariates in the model, nor are there parameter estimates. In the attached dataset for the paper, minimum and maximum (LoAge and UpAge) seem to correspond to mA and MA, but this is not clear. Further, age is not recorded in whole years, which suggests that some kind of average is being computed.   Recommendation: Refit the model with 5-year age groups modeled as either a random effect or as indicator variables. This will require redefining the outcome as counts in locations during years among different ages. However, this will actually provide much more information for policy-makers and make an important contribution to understanding the distribution of malaria across Malawi and how it varies demographically. To construct a covariate for the spatial prediction component, 5-year age data are available from WorldPop. Additionally, the authors should add a table of descriptive statistics for the covariates and outcome data. **Thank you. We have added the reference that adds detail to the methodology that transforms prevalence to the target PFPR in 2-10-year-olds.  Prevalence in 2 to 10 is what is traditionally used, and our malaria program was ok with these predictions for these ages as these would be comparable to other reports.**

 The authors note a number of advances (and challenges to malaria control). Why not include these as indicator variables in the model. For example, an indicator could be included in years 2018-2022 to indicate that PBO-based bed nets were introduced. Similarly, the time period spanning the covid pandemic could also be included as an indicator variable. Right now, all of the variability in prevalence that is due to those factors is absorbed in the random effects. Recommendation: Refit the model to account for (in some way, even if basic) the advances/setbacks in malaria control. **Thank you for your thoughtful recommendation. We have carefully considered your suggestion to refit the model to account for advances/setbacks in malaria control. Still, we have chosen to maintain the approach where the malaria control setbacks are accounted for by the random parameter in the model. Including such covariates requires that the resolution of the covariate is the same as that of the target prediction grid, which we currently do not have.**

 The references are not entirely appropriate. For example, the authors cite a paper on malaria chemotherapy from 2010 to indicate that malaria is a public health problem. Why not the WHO world malaria report that is released every year? Also, the paper is about P. falciparum malaria, but they cite a paper on P. knowlesi. Recommendation: The authors need to revisit the references and update them to be the most relevant citations **We have revised the references in the manuscript.**

# Reviewer report: Reviewer response for version 1

**DOI:** 10.21956/wellcomeopenres.21477.r63185

**Published:** 2023-08-18

**Authors:** Ebenezer Krampah Aidoo

**Affiliations:** 1 Accra Technical University, Accra, Ghana

The study used geostatistical analyses to describe malaria epidemiology in both space and time during the last **two decades / 22 years** **(clarity will be needed from authors since both are used)** to guide targeted intervention(s) in Malawi. The importance of such a study cannot be overemphasized since it sets the tone to understand past malaria transmissions and how to transition from malaria control to elimination in the future. Generally, the manuscript is well written. However, the authors can further improve the manuscript by considering these suggestions.

**Title** – Consider using **Over** *two decades of*… for the title or maintain as such if you meant 20 years and effect the changes in the manuscript to capture the actual meaning

**Introduction **

- For reference 2, consider using the 2022 malaria report
- Italicize *Plasmodium* and species names throughout the manuscript. Also, capitalize “a” in anopheles
- “ *As malaria transmission declines, its heterogeneity will increase, and transmission will be increasingly driven from ‘hotspots.*’”

**Rather** consider presenting these two scenarios below ( **which I consider as a better alternative**) with the reference

- ...transmission will be increasingly focal and at smaller scales within these foci, ‘‘hotspots’’, maintain higher malaria transmission and a consistent parasite reservoir
- Additionally, infections tend to cluster in certain demographic ‘‘hot’’ populations, or ‘‘hotpops’’, linked with demographic risk factors for transmission
  (Bousema T, Griffin JT, Sauerwein RW, Smith DL, Churcher TS, et al. (2012) Hitting hotspots: spatial targeting of malaria for control and elimination. PLoS Med 9(1): e1001165.) ^1^

**Or**

- add to your statement “driven from ‘hotspots” to ‘‘hotpops’’ and back with the relevant references
  Yangzom T, Gueye CS, Namgay R, Galappaththy GN, Thimasarn K, et al. (2012) Malaria control in Bhutan: case study of a country embarking on elimination. Malar J 11: 9. ^2^
  Chuquiyauri R, Paredes M, Penataro P, Torres S, Marin S, et al. (2012) Socio-demographics and the development of malaria elimination strategies in the low transmission setting. Acta Trop 121: 292–302. ^3^
  Wesolowski A, Eagle N, Tatem AJ, Smith DL, Noor AM, et al. (2012) Quantifying the impact of human mobility on malaria. Science 338: 267–270. ^4^
- Could the authors clarify what they mean by “ *against **clinical malaria** and 38% (95% CI 18, 53) against **severe malaria***” and also all cause hospitalization

**Results**

- “ *Figure 4 below shows the national PFPR2_10 estimates and their associated 95% confidence intervals for the years 2000 to 2022. The same estimates are presented in Table 1 below*”
- Consider deleting one (preferably Fig 4)

**D** **iscussion**

- “ *For a study that analyzed the last 20 years of available prevalence data to understand the changing transmission patterns in the 21-year period between 2000 and 2021*”, it would have been ideal to support the study with some years (not necessarily 20 years) of entomological data. The absence of entomological data limits the discussion about abundant vectors in Malawi and whether their relative distribution has changed before and after the implementation of malaria controls. Consider this as a limitation of your study
- “ *In general, the PfPR2_10 declined over the 22 years*”. However, the discussion covered 2000 – 2015. It will be ok to fill in the gap in the subsequent years what accounted for the decline
- “ *The mean modeled national PfPR2_10 in 2000 was 43.93 % (95% CI:17.9 to 73.8%) and declined to 19.2% (95%CI 7.49 to 37.0%) in 2022. The smoothened estimates of PfPR2_10 indicate that malaria prevalence is very heterogeneous with hotspot areas concentrated on the southern shores of Lake Malawi and the country's central region*”.
**Why this is the case with respect to the **southern shores of Lake Malawi and the country's central region has not been well discussed except to generalized “there are hotspots of high transmission and areas of very low transmission. This is due to varied climatic conditions, vector and parasite resistance, conducive environmental factors in urban and rural areas, and, varied intervention uptake in the different parts of the country” **. **Please elaborate on which applies to **the **southern shores of Lake Malawi and the country's central region. Heterogeneity may be informed by **malaria endemicity, urbanization, regional malaria surveillance policies, and other factors.**
- “ *Comparison of the high-resolution maps shows the spatial-temporal decline in malaria prevalence especially in Northern parts of Malawi across the years*”.
  Could the authors discuss what may be accounting for this

Is the work clearly and accurately presented and does it cite the current literature?

Yes

If applicable, is the statistical analysis and its interpretation appropriate?

I cannot comment. A qualified statistician is required.

Are all the source data underlying the results available to ensure full reproducibility?

Yes

Is the study design appropriate and is the work technically sound?

Yes

Are the conclusions drawn adequately supported by the results?

Yes

Are sufficient details of methods and analysis provided to allow replication by others?

Yes

Reviewer Expertise:

My research interests are in the areas of malaria epidemiology, host-parasite interactions, dynamics of disease transmission, antimalarial drug resistance and use of molecular tools to better understand the transmission of malaria, other infectious diseases (COVID-19, Helicobacter pylori infection etc) and neglected tropical diseases (Schistosomiasis, Soil-transmitted helminth infection etc

I confirm that I have read this submission and believe that I have an appropriate level of expertise to confirm that it is of an acceptable scientific standard, however I have significant reservations, as outlined above.

## References

[1] : Hitting Hotspots: Spatial Targeting of Malaria for Control and Elimination. *PLoS Medicine* .2012;9(1) : 10.1371/journal.pmed.1001165 10.1371/journal.pmed.1001165 PMC326943022303287

[2] : Malaria control in Bhutan: case study of a country embarking on elimination. *Malaria Journal* .2012;11(1) : 10.1186/1475-2875-11-9 10.1186/1475-2875-11-9 22230355 PMC3278342

[3] : Socio-demographics and the development of malaria elimination strategies in the low transmission setting. *Acta Tropica* .2012;121(3) : 10.1016/j.actatropica.2011.11.003 292-302 10.1016/j.actatropica.2011.11.003 PMC329404622100446

[4] : Quantifying the Impact of Human Mobility on Malaria. *Science* .2012;338(6104) : 10.1126/science.1223467 267-270 10.1126/science.1223467 PMC367579423066082

# Response

**Published:** 2023-12-22

**Authors:** Donnie Mategula

**Affiliations:** Malawi-Liverpool Wellcome Trust Clinical Research Programme, Malawi

**Dear Ebenezer Krampah Aidoo, Many thanks for your helpful and thoughtful comments. We have re-submitted an updated manuscript that addresses your suggestions and concerns. Please find our detailed responses below:**

 Title – Consider using Over two decades of… for the title or maintain as such if you meant 20 years and effect the changes in the manuscript to capture the actual meaning **Thank you for the suggestion. The two decades in the title is used loosely and not in its literal meaning.**  

 Introduction For reference 2, consider using the 2022 malaria report **The reference has been updated to the 2022 malaria report**  

 Italicize Plasmodium and species names throughout the manuscript. Also, capitalize “a” in anopheles **Plasmodium and species names have been italicized throughout the manuscript, and Anopheles is capitalized.**  

 “As malaria transmission declines, its heterogeneity will increase, and transmission will be increasingly driven from ‘hotspots.’” Rather consider presenting these two scenarios below (which I consider as a better alternative) with the reference ...transmission will be increasingly focal and at smaller scales within these foci, ‘‘hotspots’’, maintain higher malaria transmission and a consistent parasite reservoir **The suggested changes have been effected.**  

 Additionally, infections tend to cluster in certain demographic ‘‘hot’’ populations, or ‘‘hotpops’’, linked with demographic risk factors for transmission   (Bousema T, Griffin JT, Sauerwein RW, Smith DL, Churcher TS, et al. (2012) Hitting hotspots: spatial targeting of malaria for control and elimination. PLoS Med 9(1): e1001165.) 1 Or add to your statement “driven from ‘hotspots” to ‘‘hotpops’’ and back with the relevant references   Yangzom T, Gueye CS, Namgay R, Galappaththy GN, Thimasarn K, et al. (2012) Malaria control in Bhutan: case study of a country embarking on elimination. Malar J 11: 9. 2   Chuquiyauri R, Paredes M, Penataro P, Torres S, Marin S, et al. (2012) Socio-demographics and the development of malaria elimination strategies in the low transmission setting. Acta Trop 121: 292–302. 3   Wesolowski A, Eagle N, Tatem AJ, Smith DL, Noor AM, et al. (2012) Quantifying the impact of human mobility on malaria. Science 338: 267–270. 4 **Thank you for the suggestions, the text has been revised.**  

 Could the authors clarify what they mean by “against clinical malaria and 38% (95% CI 18, 53) against severe malaria” and also all cause hospitalization **This refers to uncomplicated malaria. The text has been updated**.  

 Results “Figure 4 below shows the national PFPR2_10 estimates and their associated 95% confidence intervals for the years 2000 to 2022. The same estimates are presented in Table 1 below”   Consider deleting one (preferably Fig 4) **The  table shows the exact estimates, while the figure demonstrates the trends**  

 Discussion “For a study that analyzed the last 20 years of available prevalence data to understand the changing transmission patterns in the 21-year period between 2000 and 2021”, it would have been ideal to support the study with some years (not necessarily 20 years) of entomological data. The absence of entomological data limits the discussion about abundant vectors in Malawi and whether their relative distribution has changed before and after the implementation of malaria controls. Consider this as a limitation of your study **Thank you for the suggestion, however, we are of the opinion that this would be beyond the scope of the current analysis.**  

 “In general, the PfPR2_10 declined over the 22 years”. However, the discussion covered 2000 – 2015. It will be ok to fill in the gap in the subsequent years what accounted for the decline   “The mean modeled national PfPR2_10 in 2000 was 43.93 % (95% CI:17.9 to 73.8%) and declined to 19.2% (95%CI 7.49 to 37.0%) in 2022. The smoothened estimates of PfPR2_10 indicate that malaria prevalence is very heterogeneous with hotspot areas concentrated on the southern shores of Lake Malawi and the country's central region”.   Why this is the case with respect to the southern shores of Lake Malawi and the country's central region has not been well discussed except to generalized “there are hotspots of high transmission and areas of very low transmission. This is due to varied climatic conditions, vector and parasite resistance, conducive environmental factors in urban and rural areas, and, varied intervention uptake in the different parts of the country”. Please elaborate on which applies to the southern shores of Lake Malawi and the country's central region. Heterogeneity may be informed by malaria endemicity, urbanization, regional malaria surveillance policies, and other factors.   “Comparison of the high-resolution maps shows the spatial-temporal decline in malaria prevalence especially in Northern parts of Malawi across the years”. Could the authors discuss what may be accounting for this **The authors are currently working on a study to fully understand the drivers of malaria heterogeneity in Malawi. Early findings show that this is a complex phenomenon with an interplay of several reasons such as migration, seasonal farming, suitable climatic conditions etc. It is too early to postulate on the specific causes of heterogeneity in different hotspot areas**

# Reviewer report: Reviewer response for version 1

**DOI:** 10.21956/wellcomeopenres.21477.r63176

**Published:** 2023-08-18

**Authors:** Adilson DePINA

**Affiliations:** 1 Malaria Elimination Program, CCS-SIDA / Ministry of Health, Praia, Santiago, Cape Verde; 2 Laboratório de Entomologia Médica, Instituto Nacional de Saúde Pública, Praia, Santiago, Cape Verde

1. I would recommend that the article is copy-edited.

 2. The details of methods and analysis provided could also be improved?

 3. For Statistical analyses, I can understand a lot of work done. However, it is not my background speciality, so I prefer a qualified statistician to assess it.

**General Observation**

 The manuscript is an interesting analysis of malaria data in the country. There is important data and information about the situation in the country, that could be used to sustain the malaria elimination, but also that could be used to inspire the other countries to achieve this goal.  

 Despite the pertinence of the theme, I feel that a thorough copy-edit of the article will be beneficial.

**Some points:**

- Already in the introduction we come across the verb conjugation, sometimes in the past, but also in the present. In addition, throughout the text, there are difficulties with verb conjugation. Despite the list of suggestions for improvements presented, a review by an expert on the subject and a copy-edit is necessary.
- The Plasmodium species ( *Plasmodium falciparum*) and the others, must be in italic; Please review it along the manuscript;
- The aim described in the last paragraph of the introduction is not in accordance with the methodology used and the title of the work. Please, reword it.
- What means the blue areas on the Figure 1. The Figure should be better described.
- The table 2 is too long to maintain in the main document. May it could be presented as supplementary material.
- On the Discussion part, the paragraph about the NSP history on the country may must be reviewed. It is a lot of information that doesn’t make sense regarding all data/information that we have to discuss. “ *Within this period between 2000–2022, there have been four strategic malaria plans...*" And " *The subsequent 2023 to 2030 malaria strategic plan is crucial to the elimination of malaria.*" The NSP 2023 – 2030 is already done? Consider reviewing this entire part, reducing or eliminating it and focusing the discussion on the results obtained.

**Please consider the following corrections suggestions:**

**Abstract** **:**

- " *the Monte Carlo maximum likelihood methods. District level prevalence estimates*" -- the Monte Carlo maximum likelihood method. District-level prevalence estimates *.*
- *"The mean modeled national" *- The mean modelled national
- " *highly likely associated with the scale up of control interventions*" - highly likely associated with the scale-up of control interventions.

**Introduction: **

- " *Plasmodium that has five known species that are responsible for human infection*" - Plasmodium which has five known species that are responsible for human infection
- " *targeted control efforts becomes essential to maximize*" - targeted control efforts become essential to maximize
- " *or the next seven-years (MSP 2023–2030)*" - for the next seven years (MSP 2023–2030).
- " *long-lasting insecticide treated bed nets*" - long-lasting insecticide-treated bed nets
- " *measles outbreak in 2017, polio outbreak in  2021 and cholera outbreak in 2020*" - a measles outbreak in 2017, a polio outbreak in 2021 and a cholera outbreak in 2020.
- " *management in over five year olds for hard-to-reach and high burden areas*" - management in over five-year-olds for hard-to-reach and high-burden areas
- " *reason why it is important to describe the malaria epidemiology*" - reason that it is important to describe malaria epidemiology
- " *The aim of this analysis is to describe the malaria epidemiology prevalence for the last two decades including description*" - This analysis aims to describe the malaria epidemiology prevalence for the last two decades including a report

**Methods**

***Data gathering***

- " *search in various data bases*" - search in various databases
- " *The key words used*"  -The keywords used

***Mode*** ***l*** ***description***

- " *The spatio-temporal variation in PfPR2-10 was modeled using geostatistical methods to borrow strength of information across time and space*" - The spatiotemporal variation in PfPR2-10 was modelled using geostatistical methods to borrow the strength of information across time and space
- " *Then standard geostatistical spatial-temporal model, then assumes that*" - The standard geostatistical spatial-temporal model assumes
- *"used in a model in order to*"- used in a model to capture
- " *Gaussian process for the spatio-temporal random*" - Gaussian process for the spatiotemporal random
- " *approach of Exceedance Probabilities was explored*" - approach to Exceedance Probabilities was explored
- " *relevant to policy makers*" - relevant to policymakers
- " *method sets policy relevant thresholds*" - method sets policy-relevant thresholds
- " *prevalence is exceeds a policy relevant threshold*" - prevalence exceeds a policy-relevant threshold
- " *exploratory and do not present it in this paper*" - exploratory and does not present in this paper.

***Model validation***

- *"empirical variogram methods was used"- *empirical variogram methods were used.
- *"the fitted model were ran" - *the fitted model was run
- *"there is spatial correlation in the data" -* there is a spatial correlation in the data

***Ethical considerations***

- *"The data used in the analysis was collated such that identity or exact" - *The data used in the analysis were collated such that the identity or exact
- *"obtained from the either the investigators, or from the National Malaria Control Programme" - *obtained from either the investigators or from the National Malaria Control Program

**Results**

- *"Comparison of the high-resolution" - *comparison of the high-resolution
- *"where decision making is at district level" - *where decision-making is at the district level
- *"we present district level prevalence estimates for the period 2016 to 2022, that coincides with the most" -  we present district-level prevalence estimates for the period 2016 to 2022, which coincides with the most*
- *"map in Figure 6 bellow"  - *map in Figure 6 below
- *"There is remarkable decline" - *There is a remarkable decline
- *"This include one district  (Chitipa)" - *This includes one district  (Chitipa)
- *"with prevelence estimates" - *with prevalence estimates
- *"District level PfPR estimates" - *District-level PfPR estimates
- *"Using variogram based techniques" -  *Using variogram-based techniques
- *"was tested for evidence for spatial correlation"- *was tested for evidence for spatial correlation
- *"process are shown in the Figure 7 below" -  *process are shown in Figure 7 below
- *"within the 95% confidence intervals" - *within the 95% confidence interval

**Discussion**

- *"other African countries with regard to its malaria policies" - *other African countries concerning its malaria policies
- *"the country was again first to change" -* the country was again the first to change
- *"introduction of artemisinin combination therapies" -* the introduction of artemisinin combination therapies
- *"use of indoor residual spraying, mass net distribution of long-lasting insecticide treated" - *the use of indoor residual spraying, mass net distribution of long-lasting insecticide-treated
- *"the roll out of the malaria" - *the roll-out of the malaria
- *"patterns in the 21-year period between 2000 and 2021" - *patterns in the 21 years between 2000 and 2021
- *"uncertainty at district level for the period of interest"- *uncertainty at the district level for the period of interest
- *"within this 22-year period" - *within these 22 years
- *"In the follow up years, between 2010 and 2015" - *In the follow-up years, between 2010 and 2015
- *"the decline that was observed in this period" -* the decline that was observed during this period
- *"environmental conditions, in this case temperature" - *environmental conditions, in this case, temperature
- *"that is sparse for high resolution predictions" -* that is sparse for high-resolution predictions
- *"efforts to model spatio-temporal heterogeneity"- *efforts to model spatiotemporal heterogeneity
- *"Despite this, to date this is the most detailed/ up to date description" -* Despite this, to date, this is the most detailed/up-to-date description

**Conclusion and Recommendations** ***(consider to correct the r to R)***

- *"focus on targeting of control measures in areas"* - focus on targeting control measures in areas

***Consent***

- *"The authors have assumed all original sampling have been cleared"* - The authors have assumed all original sampling has been cleared

Is the work clearly and accurately presented and does it cite the current literature?

Partly

If applicable, is the statistical analysis and its interpretation appropriate?

I cannot comment. A qualified statistician is required.

Are all the source data underlying the results available to ensure full reproducibility?

Yes

Is the study design appropriate and is the work technically sound?

Yes

Are the conclusions drawn adequately supported by the results?

Yes

Are sufficient details of methods and analysis provided to allow replication by others?

Partly

Reviewer Expertise:

Public Health, Malaria Elimination, Vector control, Program management, malaria Epidemiology

I confirm that I have read this submission and believe that I have an appropriate level of expertise to confirm that it is of an acceptable scientific standard, however I have significant reservations, as outlined above.

# Response

**Published:** 2023-12-22

**Authors:** Donnie Mategula

**Affiliations:** Malawi-Liverpool Wellcome Trust Clinical Research Programme, Malawi

**Dear Adilson DePINA, Many thanks for your helpful and thoughtful comments. We have re-submitted an updated manuscript that addresses your suggestions and concerns. Please find our detailed responses below.**

1. I would recommend that the article is copy-edited.

**Thank you. We have proofread and edited the article.**

1. The details of methods and analysis provided could also be improved?

**We have revised the methods section **  

 3. For Statistical analyses, I can understand a lot of work done. However, it is not my background speciality, so I prefer a qualified statistician to assess it.   General Observation   The manuscript is an interesting analysis of malaria data in the country. There is important data and information about the situation in the country, that could be used to sustain the malaria elimination, but also that could be used to inspire the other countries to achieve this goal.    Despite the pertinence of the theme, I feel that a thorough copy-edit of the article will be beneficial.   Some points: Already in the introduction we come across the verb conjugation, sometimes in the past, but also in the present. In addition, throughout the text, there are difficulties with verb conjugation. Despite the list of suggestions for improvements presented, a review by an expert on the subject and a copy-edit is necessary.   The Plasmodium species (Plasmodium falciparum) and the others, must be in italic; Please review it along the manuscript; **This has been reviewed**  

 The aim described in the last paragraph of the introduction is not in accordance with the methodology used and the title of the work. Please, reword it. **This has been reworded**  

 What means the blue areas on the Figure 1. The Figure should be better described. **The blue represents water specifically the major lakes in Malawi.**  

 The table 2 is too long to maintain in the main document. May it could be presented as supplementary material. **We have requested the editors to move Table 2 to supplementary material**  

 On the Discussion part, the paragraph about the NSP history on the country may must be reviewed. It is a lot of information that doesn’t make sense regarding all data/information that we have to discuss. “Within this period between 2000–2022, there have been four strategic malaria plans..." And "The subsequent 2023 to 2030 malaria strategic plan is crucial to the elimination of malaria." The NSP 2023 – 2030 is already done? Consider reviewing this entire part, reducing or eliminating it and focusing the discussion on the results obtained. **Thank you. Your comments are somewhat unclear, but parts of this section have been revised. **  

 Please consider the following corrections suggestions: **All the suggested corrections have been updated.**

# Reviewer report: Reviewer response for version 1

**DOI:** 10.21956/wellcomeopenres.21477.r61881

**Published:** 2023-08-02

**Authors:** Manuela Runge

**Affiliations:** 1 Northwestern University, Evanston, Illinois, USA

The authors present a geostatistical analysis of Malawi for the years from 2000 to 2022, which is a follow-up of a previous study that only included 2010-2017. The aim of the study is to characterize malaria prevalence in the last two decades which can inform 'setting the scene for the elimination agenda’ in Malawi. As transmission declines and country malaria control programs move towards sub-national or even sub-district level targeting of interventions, high-resolution maps across multiple years are of high value to identify areas of greatest need for intensified efforts. 

 Overall the paper is scientifically sound and well presented, however, it would benefit from further revision on a couple of unclear sentences and missing information. Below are the reviewer's comments and suggestions for the authors to address in the revised version. 

Is the work clearly and accurately presented and does it engage with the current literature?

- The choice of some references in the introduction and discussion do not seem to be the most appropriate one. Could the authors check and update these with better-fitting ones? For instance, 
  - Reference 1: should support the statement that malaria remains a public health problem to date, but the reference is from 2010 on 'A brief history of malaria chemotherapy'
  - Reference 3 and 4 are both used to support listing of known *plasmodium *species causing human malaria infection, however, the references cite a study on P.knowlesi malaria and a mathematical modelling study which are not most appropriate ones.
  - Reference 5 is very old and on the statement that *Pf *is leading cause on morbidity and mortality in children U5 an additional more recent reference would be beneficial.
  - Reference 17 [ **1**] cited in the discussion on the absence of climate anomalies which would support the direct effect of intervention scale-up and prevalence reduction is not the primary source, as Ref 17 the author's previous analysis for Malawi is also a geospatial analysis which includes the same sentence, however citing " Future climate projections for Malawi. 2017" which would not explain 2000-2017 trends? Is there a more appropriate reference?
  - Reference 20: is used in the result section to refer that between 2000 and 2022 four strategic plans have been implemented. However, the cited research is an effectiveness study on bed nets for 2018-2020. Perhaps the four strategic plans could be cited? In addition that information would be more suitable in the introduction rather than results. Instead reference 20 would be useful for supporting the interpretation of malaria burden trends 2018-2020 since the increase seems unexpected despite scale-up of interventions in that period.
  - Missing reference in “ *This is due to varied climatic conditions, vector and parasite resistance, conducive environmental factors in urban and rural areas and, varied intervention uptake in the different parts of the country.*”
- Could the authors check the color scheme in Figure 3? The color scale on the map does not seem to match the legend for the prevalence 51% to 100%. In addition, the authors may consider using the same color legend and cut-off values for prevalence maps and figures.
- Could the authors comment on why are there twice as many data points for 2009? Are these from the same survey that included more locations, or have multiple surveys been conducted in this year?
- In 2018, the country had introduced PBO nets, would be useful to know in how many areas, same for IRS, in how many areas? Since if these are only very few impacts on national trends is expected to be low. While for those specific districts, a greater impact would be expected.
- “ *There was a decline in the prevalence of malaria ages 2-10 (PfPR2_10) in the sampled locations over the period 2000 to 2022*.” This does not seem to describe the trend adequately, given the increase in prevalence in many places and prevalences remaining higher as there were in 2017. Is this a real phenomenon, or what happened between 2017 and 2018, especially in the northern districts? In addition, this sentence seems a repetition of a later sentence “In general, the PfPR2_10 was declining over the 22 years.
- The section on data gathering does not reference to earlier methods applied for i.e. Malawi 2010-2017 as in earlier work of the authors [ **1**], or even earlier data assembly methods referenced in the earlier paper. Does this imply that a separate data-gathering activity was performed for this analysis from 2000 to 2022?
- Table 2 appears too large for the paper and submission as a CSV or Excel file would also be more useful for the readers wishing to use the generated prevalence estimates.

Is the study design appropriate and does the work have academic merit?

- The study design and method applied are appropriate for generating high-resolution maps aiming to support subnational targeting utilizing the PrevMap package.
- The authors mention exceedance probability in their methods as a novel approach. It is unclear why the authors mention this approach if no results from it are included? Especially since authors describe its usefulness in setting policy-relevant thresholds, it would be great if these could be included, for the identification and validation of hot spots, and also to allow comparison to the earlier paper, that includes these maps [ **1**].
- In their work, the authors touch on some aspects that would be useful or required in future research or intervention planning, however, how their generated maps could help in these is missing. For instance, can the maps be also used to inform sub-district intervention targeting? Even if methods might be less ideal for routine data (as authors write in discussion), could there also be advantages, given that sub-district estimates will be needed (as written in the introduction), and that the methods had been applied on incidence elsewhere [ **2**]? In addition, to " *guide targeted control efforts in the remaining hotspot areas*" more robust tools are needed, what kind of tools, data, surveillance, or improved geospatial-based methods?
- The submitted work is an updated version of a previous publication **[1]**, which however is not very clear throughout the paper where more cross-referencing and comparison to the earlier version would have been expected, instead of a single sentence mentioning in the introduction. No objections to this from the reviewer if preferred by the authors, however, more transparency on these two versions which present very similar work and methods would be beneficial for the reader.

Are sufficient details of methods and analysis provided to allow replication by others?

 The methods would benefit from some clarification. If some of this has been addressed in the earlier work by the authors **[1]**, a statement would be useful that points towards where further details on methods can be found.

- Across surveys the diagnostic used for malaria infection could have varied between mRDT and microcopy, how was this accounted for?
- The provided dataset includes locations with very low sample sizes, have these been included in the analysis, or have any adjustments been made to account for low sample sizes?
- The provided dataset includes locations where the minimum age is above 10 years, while in the analysis standardized prevalence estimates 2-10 years were included. Some more details on age standardization and reference would be helpful for the reader and to ensure reproducibility. i.e. reference to [ **3**], which seemed to have been used?

If applicable, is the statistical analysis and its interpretation appropriate?

 The reviewer is not a geospatial modeling expert. Nevertheless, below are a few questions on the methodology from an epidemiological perspective for which an answer would be greatly appreciated. If some of this has been addressed in the earlier work by the authors **[1]**, a statement would be useful that points towards where the further details on methods can be found.

- Could the authors comment on handling of seasonality in survey prevalence estimates and TSI? It appears that TSI and prevalence were included at the annual level in the model, could have exclusion of information on the month of the survey at which prevalence was measured introduced a bias? Or has this been adjusted for? If so, the authors would need to include the month of survey data collection in the dataset provided in the supplement.
- Overall estimated prevalences per district for the same years as included in the previous study by the authors align quite well to the updated estimates with ~less than ~2% absolute difference, despite of TSI being included in the presented but not in earlier work. The authors may consider adding a sentence on this and the reasoning behind adding TSI to the model (as included in discussion).
- The previous study by the author's included a description of population-adjusted risk, which would also be relevant for the current work if applied, could authors add how prevalence predictions per pixel and at aggregated level were adjusted for population or where to find these details via cross-referencing?
- Could the authors add information on whether pixel-level prevalence estimates were aggregated weighted by population and using mean or median in the text? The abstract includes this but not the main text. What source for population estimates were used? 

Are all the source data underlying the results available to ensure full reproducibility?

- The source of the Temperature Suitability Index (TSI) , population estimates, and shapefiles are not mentioned or provided.
- In the dataset provided, what does a lower age of 0 mean? Are these newborns, or infants between age 0 to 1 years? If from DHS, the minimum age is 0.5 (6 months).
- If the authors could add a statement of analysis code sharing that would be useful to the readers that may want to reproduce the work. 

Are the conclusions drawn adequately supported by the results?

 Overall the conclusions are supported by the results and adequate given the malaria situation in Malawi and global trends in malaria intervention planning guidance. However, it would be great if the authors could comment on a few questions in the conclusion.

- The conclusions in this paper differ quite drastically from the author's previous conclusion of 2010-2017 prevalence predictions [ **1**]. Concluding that targeted interventions instead of universal coverage are needed. Between earlier work and this work have been several years where malaria pattern has changed, however, this change since 2017 and the change in recommendations for malaria intervention strategy in Malawi should be made clearer in the presented paper, emphasizing the observed changes in the country (as well as gllobal policy recommendations) that support the new conclusion.
  - The argument for universal coverage in earlier work has been that there would be only very few low-transmission areas. However, when looking at Figure 6, heterogeneity and low prevalence areas seemed to have decreased (instead of increased) since 2017. At high resolution, this is difficult to assess from Figure 5 (see separate comment to show fewer maps), but seems to support an increase in low prevalence areas. A supplementary figure on timelines for each of the districts would be useful to have. Further comments from the authors on these observations would be helpful, is it perhaps also a matter of thresholds?.
- The authors write in the introduction that since the Chipeta et al 2019 et al paper **[1]** “ *there have been several exciting developments in malaria control in the period between 2017 and 2021*” However these do not come up again in the discussion or conclusions.
- In the conclusion, it is written that “ *targeting of control measures in areas of highest need*. ” and the results mention heterogeneity and high prevalence areas, however, which these areas are in Malawi are not mentioned. Perhaps the authors could identify these areas by adding them to the text, or referring to the accompanying paper on stratification where the prevalence estimations were used together with other information to inform intervention targeting [ **4**].

**Typos and minor comments on format and unclear sentences**

 The authors may consider correcting the following identified issues in the text:

- In the introduction it is written that “ *NMCP is poised to develop the revised malaria strategic plan (MSP) in 2023–2030*”, however in a later sentence, the next strategic plan period is written as 2022–2027. This is confusing and would benefit from clarification. Relatedly, Malaria strategic plan 2017-2022 appears once as 2016-2022.
- In the discussion, the authors write that “ *Malaria remains a public health concern, especially in Malawi*” . However, Malawi is not one of the 'high burden to high impact' countries, and 'only' contributes 2% to the global malaria burden ( HBHI document here) ^5^. Perhaps the authors could edit the sentence to reflect this (i.e. replace especially with also).
- The display of text and figures does not seem to be in the same format and order as in the submission, and references to “above” or “below” should be changed to refer to the specific table, figure, or section instead.
- Under the model validation paragraph, ”correlation” is missing an r
- Hotspots sometimes in quotes, sometimes not
- Plasmodium and Anopheles should be in italics.
- In Figure 6 which statistical metric was used for aggregation?
- Unclear sentence: “ *level prevalence estimates for the period 2016 to 2022, that coincides with the most recent strategic plan.... There is remarkable decline in PfPR2_10 in 2017 as compared to the previous years*.” When looking at the period 2016 to 2022, there is only a single year prior to 2017. Perhaps the sentence “ *District level PfPR estimates for the period 2000 to 2022 are shown in Table 2.*” Could be moved up to clarify that results are presented for the different timeperiods.
- The sentence “ *The period 2000 to 2005 was associated with sampled locations having higher prevalence which was followed by points having a reducing PfPR2_10*.” does not seem grammatically correct, highest prevalence? or higher compared to what? In the second part “ *having a lower PfPR2to10?*” suggest rephrasing for improved clarity.
- Suggested edits for Figure 1 even if obvious and mentioned in the text, a color legend or mentioning of regions in caption would be helpful for the reader. Since the paper aims to provide subnational predictions for targeted interventions, and TSI, is the single main covariate in the model used, could Figure 1 show districts colored by TSI instead of regions, which does not provide much information?
- Figure 5 is too small, suggest to show 2016-2022 only as in Figure 6 with district boundaries (+ 2000 for baseline endemicity), this would also improve the visual transition from pixel level to aggregated estimates.
- In Figure 6, A and B are not described in the caption
- Figure 7 would benefit from clearer labelling and higher resolution, i.e. a) and b) and y-axis and x-axis labels.

Is the work clearly and accurately presented and does it cite the current literature?

Partly

If applicable, is the statistical analysis and its interpretation appropriate?

I cannot comment. A qualified statistician is required.

Are all the source data underlying the results available to ensure full reproducibility?

Partly

Is the study design appropriate and is the work technically sound?

Yes

Are the conclusions drawn adequately supported by the results?

Partly

Are sufficient details of methods and analysis provided to allow replication by others?

Partly

Reviewer Expertise:

Malaria epidemiologist and mathematical modeller of malaria intervention impact.

I confirm that I have read this submission and believe that I have an appropriate level of expertise to confirm that it is of an acceptable scientific standard, however I have significant reservations, as outlined above.

## References

[1] : Geostatistical analysis of Malawi's changing malaria transmission from 2010 to 2017. *Wellcome Open Res* .2019;4: 10.12688/wellcomeopenres.15193.2 57 10.12688/wellcomeopenres.15193.2 31372502 PMC6662685

[2] : Spatiotemporal mapping of malaria incidence in Sudan using routine surveillance data. *Sci Rep* .2022;12(1) : 10.1038/s41598-022-16706-1 14114 10.1038/s41598-022-16706-1 35982088 PMC9387890

[3] : Standardizing estimates of the Plasmodium falciparum parasite rate. *Malar J* .2007;6: 10.1186/1475-2875-6-131 131 10.1186/1475-2875-6-131 17894879 PMC2072953

[4] : Malaria Burden Stratification in Malawi- A report of a consultative workshop to inform the 2023-2030 Malawi Malaria Strategic Plan. *Wellcome Open Research* .2023;8: 10.12688/wellcomeopenres.19110.1 10.12688/wellcomeopenres.19110.1 PMC1043289037600585

[5] : High burden to high impact: a targeted malaria response. *World Health Organisation* .2018; Reference source

# Response

**Published:** 2023-12-22

**Authors:** Donnie Mategula

**Affiliations:** Malawi-Liverpool Wellcome Trust Clinical Research Programme, Malawi

**Dear Manuela Runge Many thanks for your helpful and thoughtful comments. We have re-submitted an updated manuscript that addresses your suggestions and concerns. Please find our detailed responses below.**

 The authors present a geostatistical analysis of Malawi for the years from 2000 to 2022, which is a follow-up of a previous study that only included 2010-2017. The aim of the study is to characterize malaria prevalence in the last two decades which can inform 'setting the scene for the elimination agenda’ in Malawi. As transmission declines and country malaria control programs move towards sub-national or even sub-district level targeting of interventions, high-resolution maps across multiple years are of high value to identify areas of greatest need for intensified efforts.   Overall the paper is scientifically sound and well presented, however, it would benefit from further revision on a couple of unclear sentences and missing information. Below are the reviewer's comments and suggestions for the authors to address in the revised version.   Is the work clearly and accurately presented and does it engage with the current literature? The choice of some references in the introduction and discussion do not seem to be the most appropriate one. Could the authors check and update these with better-fitting ones? For instance, Reference 1: should support the statement that malaria remains a public health problem to date, but the reference is from 2010 on 'A brief history of malaria chemotherapy' **The citation has been updated to a more appropriate reference.**

 Reference 3 and 4 are both used to support listing of known plasmodium species causing human malaria infection, however, the references cite a study on P.knowlesi malaria and a mathematical modelling study which are not most appropriate ones. **The citation has been updated to reflect a more appropriate reference.**

 Reference 5 is very old and on the statement that Pf is leading cause on morbidity and mortality in children U5 an additional more recent reference would be beneficial. **The citation has been updated to reflect more recent reference.**

 Reference 17 [1] cited in the discussion on the absence of climate anomalies which would support the direct effect of intervention scale-up and prevalence reduction is not the primary source, as Ref 17 the author's previous analysis for Malawi is also a geospatial analysis which includes the same sentence, however citing " Future climate projections for Malawi. 2017" which would not explain 2000-2017 trends? Is there a more appropriate reference? **The citation has been updated to a more appropriate reference.**

 Reference 20: is used in the result section to refer that between 2000 and 2022 four strategic plans have been implemented. However, the cited research is an effectiveness study on bed nets for 2018-2020. Perhaps the four strategic plans could be cited? In addition that information would be more suitable in the introduction rather than results. Instead reference 20 would be useful for supporting the interpretation of malaria burden trends 2018-2020 since the increase seems unexpected despite scale-up of interventions in that period. **The information on the four strategic plans has been moved to the introduction section.  Information citing reference 20 has been updated.**

 Missing reference in “This is due to varied climatic conditions, vector and parasite resistance, conducive environmental factors in urban and rural areas and, varied intervention uptake in the different parts of the country.” **The** **reference has been added**

 Could the authors check the color scheme in Figure 3? The color scale on the map does not seem to match the legend for the prevalence 51% to 100%. In addition, the authors may consider using the same color legend and cut-off values for prevalence maps and figures. **The color scheme matches the legend for the 51% to 100% prevalence but because of overlaying points appears darker in the 2000-2005 map.**

 Could the authors comment on why are there twice as many data points for 2009? Are these from the same survey that included more locations, or have multiple surveys been conducted in this year? **There were more sampled locations in 2009, this has been included in the text.**

 In 2018, the country had introduced PBO nets, would be useful to know in how many areas, same for IRS, in how many areas? Since if these are only very few impacts on national trends is expected to be low. While for those specific districts, a greater impact would be expected. **PBO nets were 28% of the total nets distributed and were distributed in 10 districts with high malaria transmission and increased pyrethroid resistance  e.g. Machinga district that registered a decline in malaria prevalence from 20% in 2018 to 13% in 2022. This has been included in the text.**

 “There was a decline in the prevalence of malaria ages 2-10 (PfPR2_10) in the sampled locations over the period 2000 to 2022.” This does not seem to describe the trend adequately, given the increase in prevalence in many places and prevalences remaining higher as there were in 2017. Is this a real phenomenon, or what happened between 2017 and 2018, especially in the northern districts? In addition, this sentence seems a repetition of a later sentence “In general, the PfPR2_10 was declining over the 22 years. **The sentence has been revised**

 The section on data gathering does not reference to earlier methods applied for i.e. Malawi 2010-2017 as in earlier work of the authors [1], or even earlier data assembly methods referenced in the earlier paper. Does this imply that a separate data-gathering activity was performed for this analysis from 2000 to 2022? **The database that was used in the previous publication was updated.**

 Table 2 appears too large for the paper and submission as a CSV or Excel file would also be more useful for the readers wishing to use the generated prevalence estimates. **We have requested the editors to move the table to the supplementary files.**

 Is the study design appropriate and does the work have academic merit? The study design and method applied are appropriate for generating high-resolution maps aiming to support subnational targeting utilizing the PrevMap package.   The authors mention exceedance probability in their methods as a novel approach. It is unclear why the authors mention this approach if no results from it are included? Especially since authors describe its usefulness in setting policy-relevant thresholds, it would be great if these could be included, for the identification and validation of hot spots, and also to allow comparison to the earlier paper, that includes these maps [1]. **We have used confidence intervals to measure uncertainty, and we feel that two measures of uncertainty are unnecessary.**

 In their work, the authors touch on some aspects that would be useful or required in future research or intervention planning, however, how their generated maps could help in these is missing. For instance, can the maps be also used to inform sub-district intervention targeting? Even if methods might be less ideal for routine data (as authors write in discussion), could there also be advantages, given that sub-district estimates will be needed (as written in the introduction), and that the methods had been applied on incidence elsewhere [2]? In addition, to "guide targeted control efforts in the remaining hotspot areas" more robust tools are needed, what kind of tools, data, surveillance, or improved geospatial-based methods? **Thank you for the comment. While district level prevalence estimates are presented, the predictions made in the model are to 1 kilometer square grid. This essentially means that we can, in principle, produce estimates at any level from 1x 1 km. In the present analysis, we focus on the district level as it was the relevant one at that point in time. Future work is focusing on sub-district estimates.**

 The submitted work is an updated version of a previous publication [1], which however is not very clear throughout the paper where more cross-referencing and comparison to the earlier version would have been expected, instead of a single sentence mentioning in the introduction. No objections to this from the reviewer if preferred by the authors, however, more transparency on these two versions which present very similar work and methods would be beneficial for the reader. **Thank you for the comment. We can confirm that the methods used are similar; however, while the two papers may be similar, the objectives were slightly different. In the present paper, we produce yearly estimates over two decades. That was not the main focus of the previous work as it only presented estimates for two time points. We prefer the approach used in the current paper.**

 Are sufficient details of methods and analysis provided to allow replication by others?   The methods would benefit from some clarification. If some of this has been addressed in the earlier work by the authors [1], a statement would be useful that points towards where further details on methods can be found. **In both papers, we used model-based geostatistics. A reference statement and citation of where the full description of the methods can be found has been provided.**

 Across surveys, the diagnostic used for malaria infection could have varied between mRDT and microcopy; how was this accounted for? **Thank you. Indeed, this could have varied, but this is resolved since the target predictions are transformed to the standardized format of PfPR2_10.**

 The provided dataset includes locations with very low sample sizes. Have these been included in the analysis, or have any adjustments been made to account for low sample sizes? **Low sample sizes affect the uncertainty of the estimates, which is reflected in the confidence intervals**

 The provided dataset includes locations where the minimum age is above 10 years, while in the analysis standardized prevalence estimates 2-10 years were included. Some more details on age standardization and reference would be helpful for the reader and to ensure reproducibility. i.e. reference to [3], which seemed to have been used? **A reference to the standardization has been provided.**

 If applicable, is the statistical analysis and its interpretation appropriate?   The reviewer is not a geospatial modeling expert. Nevertheless, below are a few questions on the methodology from an epidemiological perspective for which an answer would be greatly appreciated. If some of this has been addressed in the earlier work by the authors [1], a statement would be useful that points towards where the further details on methods can be found. **Thank you.We have added the reference where further details of the methods can be found**

 Could the authors comment on handling of seasonality in survey prevalence estimates and TSI? It appears that TSI and prevalence were included at the annual level in the model, could have exclusion of information on the month of the survey at which prevalence was measured introduced a bias? Or has this been adjusted for? If so, the authors would need to include the month of survey data collection in the dataset provided in the supplement. **Seasonality is accounted for primarily by the random effects parameter in the model. Additionally, most of the surveys are done during the peak malaria season which have been fairly the same over the period of interest. **  

 Overall estimated prevalences per district for the same years as included in the previous study by the authors align quite well to the updated estimates with ~less than ~2% absolute difference, despite of TSI being included in the presented but not in earlier work. The authors may consider adding a sentence on this and the reasoning behind adding TSI to the model (as included in discussion). **In the discussion, the reasons for adding TSI have been included.**

 The previous study by the author's included a description of population-adjusted risk, which would also be relevant for the current work if applied, could authors add how prevalence predictions per pixel and at aggregated level were adjusted for population or where to find these details via cross-referencing? **All the predictions made were population-adjusted.**

 Could the authors add information on whether pixel-level prevalence estimates were aggregated weighted by population and using mean or median in the text? The abstract includes this but not the main text. What source for population estimates were used? Are all the source data underlying the results available to ensure full reproducibility? The source of the Temperature Suitability Index (TSI) , population estimates, and shapefiles are not mentioned or provided. **We have added the sources of these in the manuscript**

 In the dataset provided, what does a lower age of 0 mean? Are these newborns, or infants between age 0 to 1 years? If from DHS, the minimum age is 0.5 (6 months). **It means newborns. Though not a lot, the data sources are not just MIS/DHS**

 If the authors could add a statement of analysis code sharing that would be useful to the readers that may want to reproduce the work. **Model-based geostatistics as a methodology is freely and publicly available and we have provided the reference. We would be more than happy to share the code if requested**  

 Are the conclusions drawn adequately supported by the results?   Overall the conclusions are supported by the results and adequate given the malaria situation in Malawi and global trends in malaria intervention planning guidance. However, it would be great if the authors could comment on a few questions in the conclusion. The conclusions in this paper differ quite drastically from the author's previous conclusion of 2010-2017 prevalence predictions [1]. Concluding that targeted interventions instead of universal coverage are needed. Between earlier work and this work have been several years where malaria pattern has changed, however, this change since 2017 and the change in recommendations for malaria intervention strategy in Malawi should be made clearer in the presented paper, emphasizing the observed changes in the country (as well as gllobal policy recommendations) that support the new conclusion. **Global recommendations are in full support of targeted interventions, this has also been emphasized in the malaria new malaria strategic plan for malaria elimination**

 The argument for universal coverage in earlier work has been that there would be only very few low-transmission areas. However, when looking at Figure 6, heterogeneity and low prevalence areas seemed to have decreased (instead of increased) since 2017. At high resolution, this is difficult to assess from Figure 5 (see separate comment to show fewer maps), but seems to support an increase in low prevalence areas. A supplementary figure on timelines for each of the districts would be useful to have. Further comments from the authors on these observations would be helpful, is it perhaps also a matter of thresholds?. **The authors are currently working with the malaria program on malaria microstratification. This is request is beyond the scope of this paper but will be dealt with by the authors**  

 he authors write in the introduction that since the Chipeta et al 2019 et al paper [1] “there have been several exciting developments in malaria control in the period between 2017 and 2021” However these do not come up again in the discussion or conclusions. **These have been included in the discussion.**

 In the conclusion, it is written that “targeting of control measures in areas of highest need. ” and the results mention heterogeneity and high prevalence areas, however, which these areas are in Malawi are not mentioned. Perhaps the authors could identify these areas by adding them to the text, or referring to the accompanying paper on stratification where the prevalence estimations were used together with other information to inform intervention targeting [4]. Typos and minor comments on format and unclear sentences   The authors may consider correcting the following identified issues in the text: In the introduction it is written that “NMCP is poised to develop the revised malaria strategic plan (MSP) in 2023–2030”, however in a later sentence, the next strategic plan period is written as 2022–2027. This is confusing and would benefit from clarification. Relatedly, Malaria strategic plan 2017-2022 appears once as 2016-2022. **Thank you for observing this. We have made the corrections**

 In the discussion, the authors write that “Malaria remains a public health concern, especially in Malawi” . However, Malawi is not one of the 'high burden to high impact' countries, and 'only' contributes 2% to the global malaria burden (HBHI document here) 5. Perhaps the authors could edit the sentence to reflect this (i.e. replace especially with also). **Malaria is within the top five diseases with the most morbidity and mortality; as such, it remains a public health burden in Malawi with an estimated 4.4 million cases reported in 2020.**

 The display of text and figures does not seem to be in the same format and order as in the submission, and references to “above” or “below” should be changed to refer to the specific table, figure, or section instead. **This has been done**

 Under the model validation paragraph, ”correlation” is missing an r **This has been done**

 Hotspots sometimes in quotes, sometimes not   Plasmodium and Anopheles should be in italics. **This has been done**

 In Figure 6 which statistical metric was used for aggregation? **We used means.**

 Unclear sentence: “ level prevalence estimates for the period 2016 to 2022, that coincides with the most recent strategic plan.... There is remarkable decline in PfPR2_10 in 2017 as compared to the previous years.” When looking at the period 2016 to 2022, there is only a single year prior to 2017. Perhaps the sentence “District level PfPR estimates for the period 2000 to 2022 are shown in Table 2.” Could be moved up to clarify that results are presented for the different timeperiods.  We have revised the sentence to refer to the previous year 2016. The sentence “The period 2000 to 2005 was associated with sampled locations having higher prevalence which was followed by points having a reducing PfPR2_10.” does not seem grammatically correct, highest prevalence? or higher compared to what? In the second part “having a lower PfPR2to10?” suggest rephrasing for improved clarity. **We have reworked this part for clarity.**

 Suggested edits for Figure 1 even if obvious and mentioned in the text, a color legend or mentioning of regions in caption would be helpful for the reader. Since the paper aims to provide subnational predictions for targeted interventions, and TSI, is the single main covariate in the model used, could Figure 1 show districts colored by TSI instead of regions, which does not provide much information?   Figure 5 is too small, suggest to show 2016-2022 only as in Figure 6 with district boundaries (+ 2000 for baseline endemicity), this would also improve the visual transition from pixel level to aggregated estimates.  The theme of the paper is to show the trend over the 22 years. We are happy to share the yearly rasters for readers who want details of specific years In Figure 6, A and B are not described in the caption **We have added the caption in the text.**
